# *Toxoplasma gondii* requires its plant-like heme biosynthesis pathway for infection

**DOI:** 10.1371/journal.ppat.1008499

**Published:** 2020-05-14

**Authors:** Amy Bergmann, Katherine Floyd, Melanie Key, Carly Dameron, Kerrick C. Rees, L. Brock Thornton, Daniel C. Whitehead, Iqbal Hamza, Zhicheng Dou

**Affiliations:** 1 Department of Biological Sciences, Clemson University, Clemson, South Carolina, United States of America; 2 Department of Chemistry, Clemson University, Clemson, South Carolina, United States of America; 3 Eukaryotic Pathogens Innovation Center, Clemson University, Clemson, South Carolina, United States of America; 4 Department of Animal and Avian Sciences, University of Maryland, College Park, Maryland, United States of America; 5 Department of Cell Biology and Molecular Genetics, University of Maryland, College Park, Maryland, United States of America; University of Wisconsin Medical School, UNITED STATES

## Abstract

Heme, an iron-containing organic ring, is essential for virtually all living organisms by serving as a prosthetic group in proteins that function in diverse cellular activities ranging from diatomic gas transport and sensing, to mitochondrial respiration, to detoxification. Cellular heme levels in microbial pathogens can be a composite of endogenous *de novo* synthesis or exogenous uptake of heme or heme synthesis intermediates. Intracellular pathogenic microbes switch routes for heme supply when heme availability fluctuates in their replicative environment throughout infection. Here, we show that *Toxoplasma gondii*, an obligate intracellular human pathogen, encodes a functional heme biosynthesis pathway. A chloroplast-derived organelle, termed apicoplast, is involved in heme production. Genetic and chemical manipulation revealed that *de novo* heme production is essential for *T*. *gondii* intracellular growth and pathogenesis. Surprisingly, the herbicide oxadiazon significantly impaired *Toxoplasma* growth, consistent with phylogenetic analyses that show *T*. *gondii* protoporphyrinogen oxidase is more closely related to plants than mammals. This inhibition can be enhanced by 15- to 25-fold with two oxadiazon derivatives, lending therapeutic proof that *Toxoplasma* heme biosynthesis is a druggable target. As *T*. *gondii* has been used to model other apicomplexan parasites, our study underscores the utility of targeting heme biosynthesis in other pathogenic apicomplexans, such as *Plasmodium spp*., *Cystoisospora*, *Eimeria*, *Neospora*, and *Sarcocystis*.

## Author summary

*Toxoplasma gondii* infects essentially all warm-blooded animals due to its broad species and tissue tropism. Almost one-third of the human population carry *Toxoplasma* infection, which can cause severe morbidity and mortality in immunocompromised individuals. The current antibiotics against *Toxoplasma* trigger strong side effects in some groups of patients and have limited efficacy on congenital toxoplasmosis. Thus, an urgent need for novel therapeutics exist. Here, we show that *Toxoplasma gondii* actively produces heme, a key nutrient for many subcellular activities, via its plant-like heme biosynthesis pathway for intracellular growth and acute virulence. We found that several herbicidal heme biosynthesis inhibitors and their derivatives show inhibitory effects against intracellular *Toxoplasma* growth. Our findings provide evidence that disrupting heme production in *Toxoplasma* could be an effective therapeutic strategy to control infection.

## Introduction

**Fig 1:**
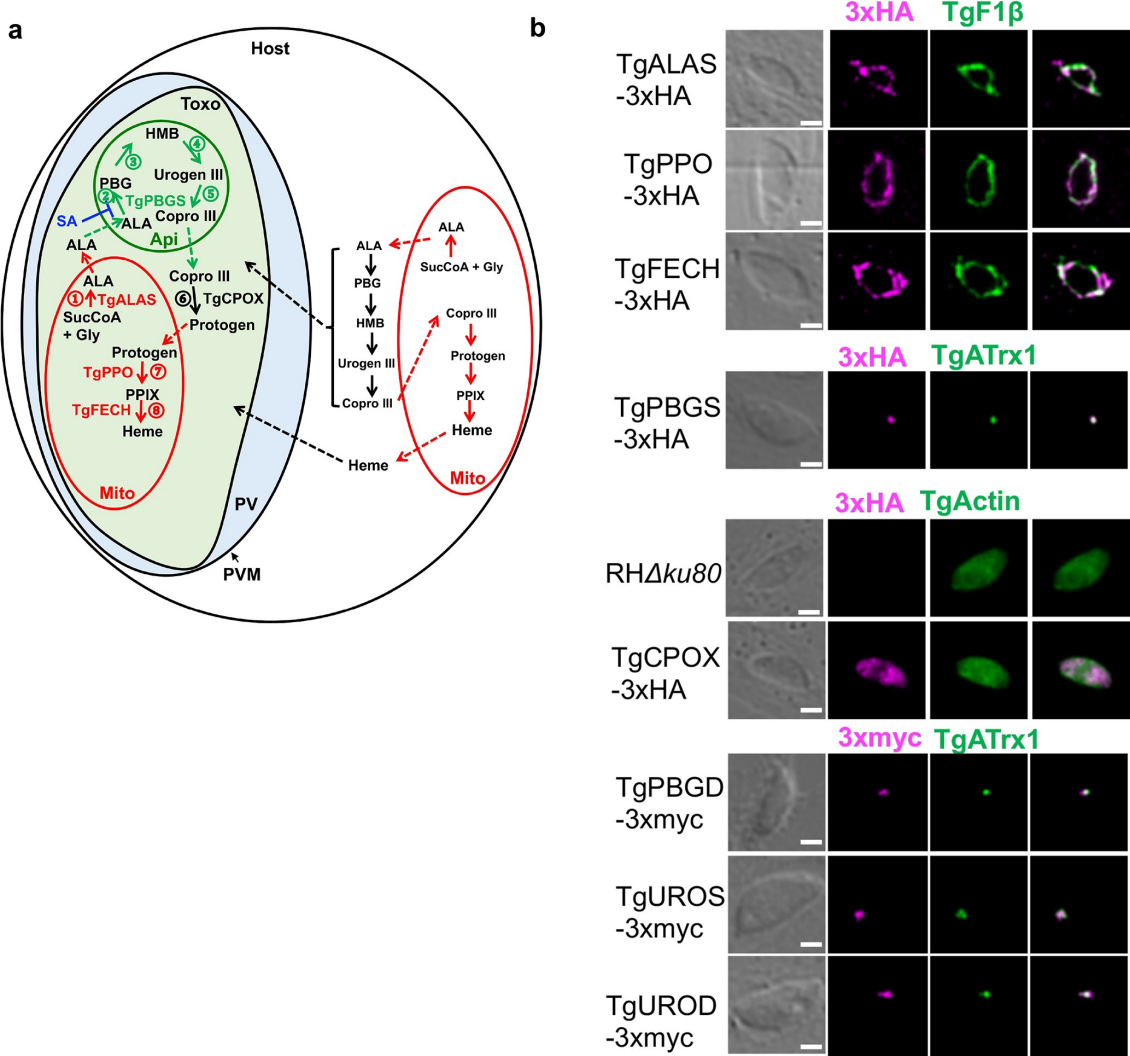
*Toxoplasma gondii* encodes its *de novo* heme biosynthetic pathway within 3 subcellular locations. **a,** The working model of the *de novo* heme biosynthesis in *Toxoplasma* parasites. The enzymes catalyzing the *de novo* heme biosynthesis are distributed within three subcellular locations in the parasites, whereas they are only localized in the mitochondria and cytoplasm in mammals. **b,** Determination of the expression of the heme biosynthetic genes in *Toxoplasma* during its acute infection and their subcellular locations by endogenous gene tagging with 3xHA or 3xmyc epitopes. A subunit of *Toxoplasma* mitochondrial ATPase (TgF1β) and an apicoplast-associated thioredoxin family protein (TgATrx1) were used as the mitochondrial and apicoplast markers, respectively. TgActin was used as a cytoplasm marker. Bar = 2 μm. ALA, 5-aminolevulinic acid; ALAS, 5-aminolevulinic acid synthase; Api, apicoplast; Copro III, coproporphyrinogen III; CPOX, Coproporphyrinogen III oxidase; FECH, Ferrochelatase; Gly, glycine; HMB, hydroxymethylbilan; IVN, intravacuolar network; Mito, mitochondria; PBG, porphobilinogen; PBGD, Porphobilinogen deaminase; PBGS, Porphobilinogen synthase; PPIX, protoporphyrin IX; PPO, Protoporphyrinogen oxidase; Protogen, protoporphyrinogen IX; PV, parasitophorous vacuole; PVM, parasitophorous vacuole membrane; SucCoA, Succinyl-CoA; Toxo, *Toxoplasma gondii*; UROD, Uroporphyrinogen III decarboxylase; Urogen III, uroporphyrinogen III; UROS, Uroporphyrinogen III Synthase.

Human protozoan pathogens share some common nutrient metabolism pathways with their counterparts in the host but show distinct features. For example, two well-known representative species within the Apicomplexa Phylum, *Toxoplasma gondii* and *Plasmodium spp*., encode a complete heme biosynthesis pathway in their genomes [1] (Fig 1A and S1 Table). The eight heme biosynthetic enzymes residing within this pathway are delivered to three subcellular locations [1–4], including the mitochondrion, cytoplasm, and apicoplast. The apicoplast is a remnant chloroplast and specifically exists in apicomplexan parasites. Heme typically serves as a prosthetic group in proteins, such as cytochromes, which play an essential role in mitochondrial respiration through the electron transport chain in parasites [5,6]. In addition, *Toxoplasma* encodes orthologs of two other hemoproteins, cytochrome P450 and catalase, in its genome, suggesting that heme is likely involved in detoxification in *Toxoplasma*. In contrast, the malaria parasites lack both genes [1].

The previous studies have successfully expressed active recombinant *Toxoplasma* porphobilinogen synthase (TgPBGS), the second enzyme residing within the parasite’s heme biosynthesis pathway, in *E*. *coli*, and solved its three-dimensional structure [7,8]. Moreover, succinylacetone (SA), an inhibitor targeting TgPBGS activity, was shown to suppress intracellular *Toxoplasma* growth at a half maximal inhibitory concentration (IC_50_) of ~ 2 mM, which sheds light on the therapeutic potential of targeting the heme biosynthetic pathway against toxoplasmosis [2]. A recent genome-wide CRISPR screen in *Toxoplasma* calculated the fitness scores of all eight heme biosynthetic genes to be below -2.7, indicating that the heme biosynthesis pathway is critical in parasite growth [9]. However, it still remains unknown whether the entire pathway is active for heme production during *Toxoplasma* infections and the extent to which *Toxoplasma* relies on this pathway for its infection.

As an intracellular parasite, *Toxoplasma* utilizes the host plasma membrane to create its own membrane-bound compartment for intracellular replication, termed the parasitophorous vacuole (PV) [10]. The PV membrane (PVM) is permeable to small solutes. Studies have demonstrated that putative nutrient pores exist on the PVM, which allow small substances with molecular weights less than ~1,300 Da to diffuse into the PV [11]. While heme molecules are significantly smaller than 1,300 Da, free heme, however, is toxic and therefore unlikely to be found in a free form. Instead, they are loosely associated with proteins or small ligands that collectively constitute the cellular heme labile pool within mammalian cells [12–14]. Therefore, it is unlikely that *Toxoplasma* is able to acquire free heme from the host cells via putative nutrient pores. Since *Toxoplasma* can ingest and digest host proteins to support its growth [15], *Toxoplasma* may acquire heme through liberating it from the digestion of the host’s hemoproteins. Collectively, both the parasite’s *de novo* heme production and/or heme acquisition from the host may contribute to parasite replication and infection.

Interestingly, the *de novo* heme biosynthesis pathway exhibits diverse patterns within the Apicomplexa Phylum. By ortholog search, several other human and animal pathogens besides *Toxoplasma* and *Plasmodium*, including *Cyclospora cayetanensis*, *Cystoisospora suis*, *Eimeria tenella*, *Hammondia hammondi*, *Neospora caninum*, and *Sarcocystis neurona*, encode a complete or a partial heme biosynthetic pathway (S1 Table). Orthologs missing for some heme biosynthetic genes could be due to incomplete coverage of genome sequencing or diverse primary sequences of these orthologs in apicomplexan parasites. For example, a UROS ortholog is not identified in the *Plasmodium* genome by primary sequence alignment with annotated UROS proteins, but a recent bioinformatic study reported a putative *Plasmodium* UROS ortholog with low similarity to annotated UROS [16]. In addition, the enzymatic activity of UROS has been observed in recombinant *Plasmodium* PBGD [17], suggesting its unique dual roles in the heme biosynthesis within *Plasmodium*. In contrast, *Babesia*, *Theileria*, and *Cryptosporidium spp*., completely lack the intact pathway [3], suggesting that these apicomplexan parasites have to scavenge heme from the host. Moreover, malaria parasites switch their requirement for *de novo* heme production at different infection stages. The *de novo* heme biosynthesis is dispensable in the blood-stage infection of *Plasmodium falciparum* [4,18], but is required for its liver-stage infection [19,20], suggesting that intracellular pathogens could switch their heme requirements based on heme availability.

## Results and discussion

To test whether all 8 genes residing within *Toxoplasma*’s *de novo* heme biosynthesis pathway are expressed in the acute infection stage, we endogenously inserted epitope tags at their C-termini (S1A Fig). Immunoblotting revealed their active expression during acute toxoplasmosis (S1C Fig). In addition, fluorescence localization experiments confirmed that *Toxoplasma* distributes its heme biosynthesis throughout the mitochondrion, cytoplasm, and apicoplast (Fig 1B, S1B Fig, and S1 Text). Overall, our findings revealed that *Toxoplasma* maintains *de novo* heme biosynthetic components during acute infection.

**Fig 2:**
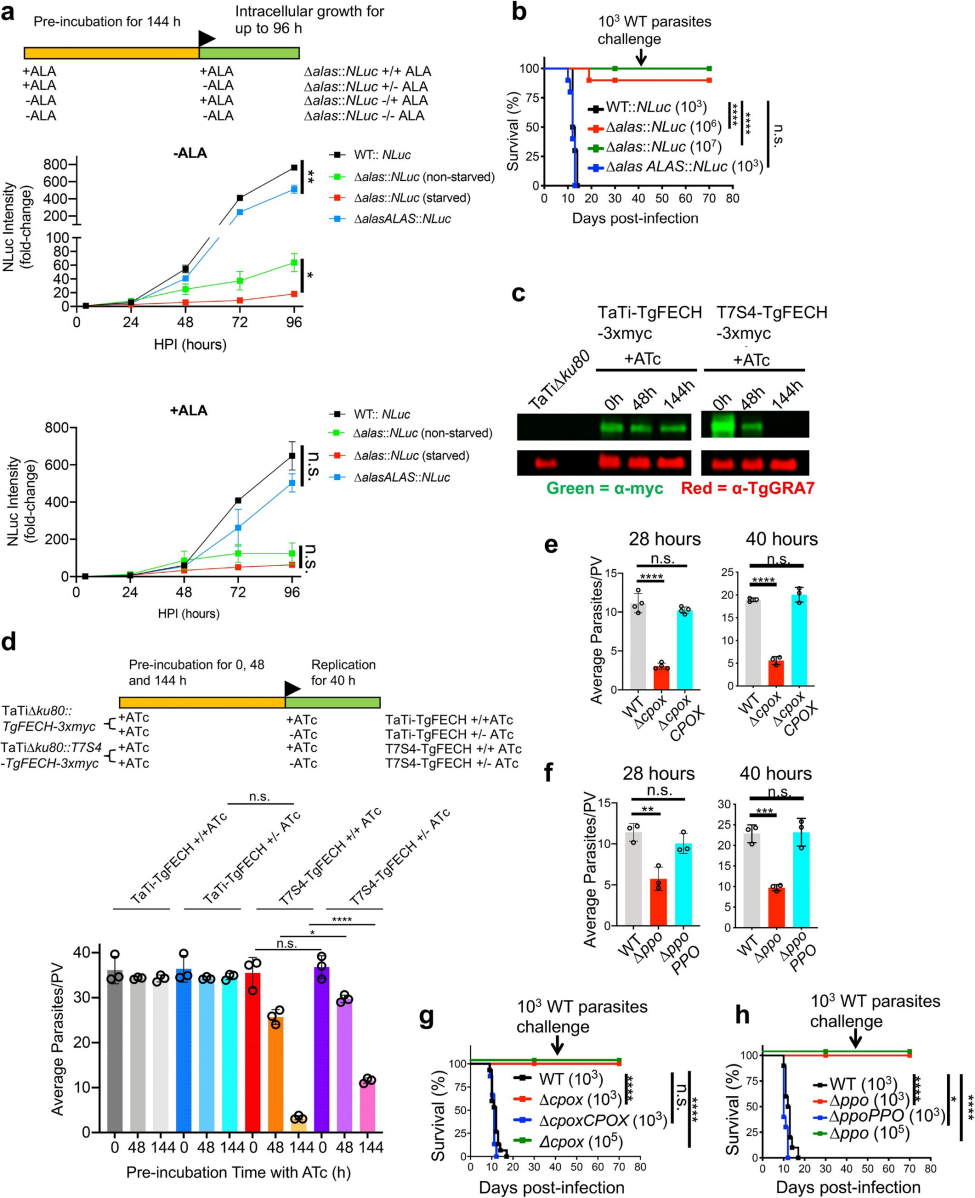
*Toxoplasma* parasites principally rely on their *de novo* heme biosynthesis for intracellular growth and pathogenesis. **a,** Growth comparison of the ALA-starved and non-starved Δ*alas*::*NLuc* parasites in media containing or lacking ALA. The parasites were grown in confluent HFFs and their luciferase activities were measured every 24 h for up to 96 h. Data represent mean ± SEM of n = 3 biological replicates. **b,** Acute virulence determination of *TgALAS*-deficient parasites in a murine model. Ten mice of equal numbers of males and females were used for each strain. **c,** Evaluation of repression efficiency of TgFECH by ATc treatment via immunoblotting. *TgFECH* was endogenously tagged with a 3xmyc tag at its C-terminus for recognition by immunoblotting. The lysates were also probed against TgGRA7 as a loading control. **d,** Replication assessment of the *TgFECH* knockdown parasites. T7S4-TgFECH and its parental strains were pre-treated with ATc for the period described in the scheme before replication assay. Data represent mean ± SD of n = 3 biological replicates. **e-f,** Replication assay of Δ*cpox* and Δ*ppo* parasites. Data represent mean ± SD of n = 3–4 biological replicates. **g-h,** Acute virulence measurement of Δ*cpox* and Δ*ppo* parasites in a murine model. 5 male and 5 female mice were used for each strain. The statistical significance for each animal study in **b, g,** and **h** was calculated using the Log-rank (Mantel-Cox) test. Statistical significance in the rest of the studies was calculated by two-tailed unpaired Student’s *t*-test. *, *p*<0.05; **, *p*<0.01; ***, *p*<0.001; ****, *p*<0.0001; n.s., not significant.

Given the possibility that *Toxoplasma* could rely on its *de novo* heme production or scavenge heme or its intermediates from the host to support infection, we deleted 5-aminolevulinic acid (ALA) synthase (*TgALAS*, TGGT1_258690), the first enzyme in the pathway, in NanoLuc luciferase-expressing wildtype (WT::*NLuc*) *Toxoplasma*. Disruption of *TgALAS* will specifically block the parasite’s *de novo* heme biosynthesis but maintain the downstream pathway intact for possible utilization of host-derived heme intermediates (Fig 1A). Generation of Δ*alas* in standard growth medium was unsuccessful until the medium was supplemented with 300 μM ALA, the product of TgALAS, suggesting that the *de novo* heme production is essential for parasite infection. To test this, we evaluated the growth of the resulting Δ*alas*::*NLuc* mutant after it was starved in ALA-free medium for 144 h. The pre-starved Δ*alas*::*NLuc* exhibited severe growth defects compared to WT::*NLuc* and Δ*alasALAS*::*NLuc* (a *TgALAS* complementation strain), and also grew more slowly relative to the non-starved Δ*alas*::*NLuc* parasites in the medium lacking ALA (Fig 2A), suggesting that the stored heme reserve within parasites enhances their intracellular growth when the parasites encounter insufficient heme production. In contrast, the pre-starved Δ*alas*::*NLuc* mutant showed comparable growth compared to the non-starved Δ*alas*::*NLuc* when they were grown in the ALA-containing medium (Fig 2A), indicating that the parasites can quickly respond to extracellular ALA for heme production. The pre-starved Δ*alas*::*NLuc* mutant displayed an extremely slow growth rate when it was grown in the ALA-free medium (Fig 2A), suggesting that the parasites may incorporate a residual level of heme and/or heme synthesis intermediates from the host. Similarly, we observed that the Δ*alas*::*NLuc* parasites showed severe defects in intracellular replication (S3A Fig) and plaque development (S3B Fig). The addition of 300 μM ALA in the medium enhanced the replication of *TgALAS*-deficient parasites regardless of pre-starvation status (S3A Fig). We also observed that the plaque formation of Δ*alas*::*NLuc* was partially restored in the medium supplemented with 300 μM ALA (S3B Fig and S1 Text). The addition of extracellular ALA could result in enhanced production and accumulation of toxic porphyrin intermediates in host cells and infected parasites [6], which may impair intracellular parasite growth. To test whether ALA can boost parasite growth to a greater extent at ALA concentrations below 300 μM, we compared the growth rates of Δ*alas*::*NLuc* in media containing 300, 100, 33.3, 11.1, and 3.7 μM ALA. Our data showed that the growth of Δ*alas*::*NLuc* was enhanced in the media supplemented with 100 μM and 300 μM ALA, and exhibited a higher increase under 300 μM ALA than 100 μM ALA (S3C Fig). This observation suggests that *Toxoplasma* parasites cannot easily access extracellular ALA, probably because ALA needs to cross multiple membranes to reach the parasite’s mitochondrion. To test the role of TgALAS in parasite acute virulence, we subcutaneously injected the Δ*alas*::*NLuc* mutant along with WT::*NLuc* and Δ*alasALAS*::*NLuc* strains into CD-1 mice and did not observe mortality in the mice infected with the Δ*alas*::*NLuc* strain, even when its inocula were 10^3^- and 10^4^-fold higher than that required for WT parasites to establish a lethal infection (Fig 2B). As expected, the infections derived from WT::*NLuc* and Δ*alasALAS*::*NLuc* strains were lethal at 10–12 days post-infection (Fig 2B). The parasite infection in the surviving mice was confirmed by seroconversion and their resistance to subsequent challenge with WT parasites.

To further confirm that the heme biosynthesis pathway is crucial in *Toxoplasma*, we attempted to ablate the ferrochelatase (*TgFECH*, TGGT1_258650), which catalyzes the last step in heme production. We detected the correct integration of the drug resistance cassette into the *TgFECH* locus (S2D Fig) but were unable to generate the straight Δ*fech* knockout, even upon supplementing the medium with 10 μM heme. Alternatively, we created a *TgFECH* knockdown strain using a tetracycline-controlled TET-OFF system. To recognize the targeted *TgFECH*, we endogenously tagged its C-terminus with a 3xmyc epitope before replacing its cognate promoter with a T7S4 promoter, a hybrid of the promoter of *Toxoplasma SAG4* (surface antigen 4) gene and the anhydrotetracycline (ATc)-responsive promoter (S4A Fig). The expression of *TgFECH* in the resulting knockdown strain, named T7S4-TgFECH-3xmyc, was below the limit of detection after 144-h treatment of ATc (Fig 2C), and the *TgFECH-*deficient mutant showed a drastic replication defect (Fig 2D). Interestingly, if the *TgFECH* repression was halted during the replication assay by removing ATc, T7S4-TgFECH-3xmyc parasites significantly increased their replication rate compared to those grown in ATc-containing medium, validating the key role of the *de novo* heme biosynthesis in parasite intracellular replication (Fig 2D). Additionally, we individually deleted coproporphyrinogen oxidase (*TgCPOX*, TGGT1_223020) and protoporphyrinogen oxidase (*TgPPO*, TGGT1_272490) within the pathway. The *TgCPOX*- and *TgPPO*-lacking mutants (Δ*cpox* and Δ*ppo*, respectively) displayed approximately 75% and 50% reduction in replication, respectively, compared to WT (Fig 2E and 2F). A luciferase-based assay also confirmed that both knockout mutants showed severe growth defects compared to WT parasites (S5A and S5B Fig). Both mutants showed significant defects in plaque formation as well (S5C and S5D Fig and S1 Text). The deletion of *TgCPOX* or *TgPPO* also resulted in the complete loss of acute virulence in *Toxoplasma* (Fig 2G and 2H). Given that TgPPO is an oxidase, such an oxidation reaction can occur even without enzymatic catalysis, albeit at lower reaction rates, which could explain why the growth defect shown in the Δ*ppo* mutant is not as severe as that of *TgALAS* and *TgFECH*-deficient parasites. These findings are also consistent with *S*. *cerevisiae*, wherein PPO is the only non-essential protein within its heme biosynthesis pathway [21]. In contrast, CPOX converts coproporphyrinogen III into protoporphyrinogen IX via decarboxylation. Given that the non-enzymatic decarboxylation rate is extremely slow [22], a spontaneous conversion is unlikely in Δ*cpox* parasites. Instead, *Toxoplasma* encodes an oxygen-independent coproporphyrinogen dehydrogenase (*TgCPDH*, TGGT1_288640), which may also help bypass the reaction catalyzed by TgCPOX to sustain partial *de novo* heme production in the parasites, albeit possibly at a lower level. Collectively, the systematic phenotypic characterization of a series of mutants lacking functional *de novo* heme production in *Toxoplasma* established its crucial importance in parasite growth and pathogenesis. Although *Toxoplasma* may acquire heme or its intermediates from the host via a salvage pathway, it is not sufficient for supporting normal parasite growth and acute virulence.

**Fig 3:**
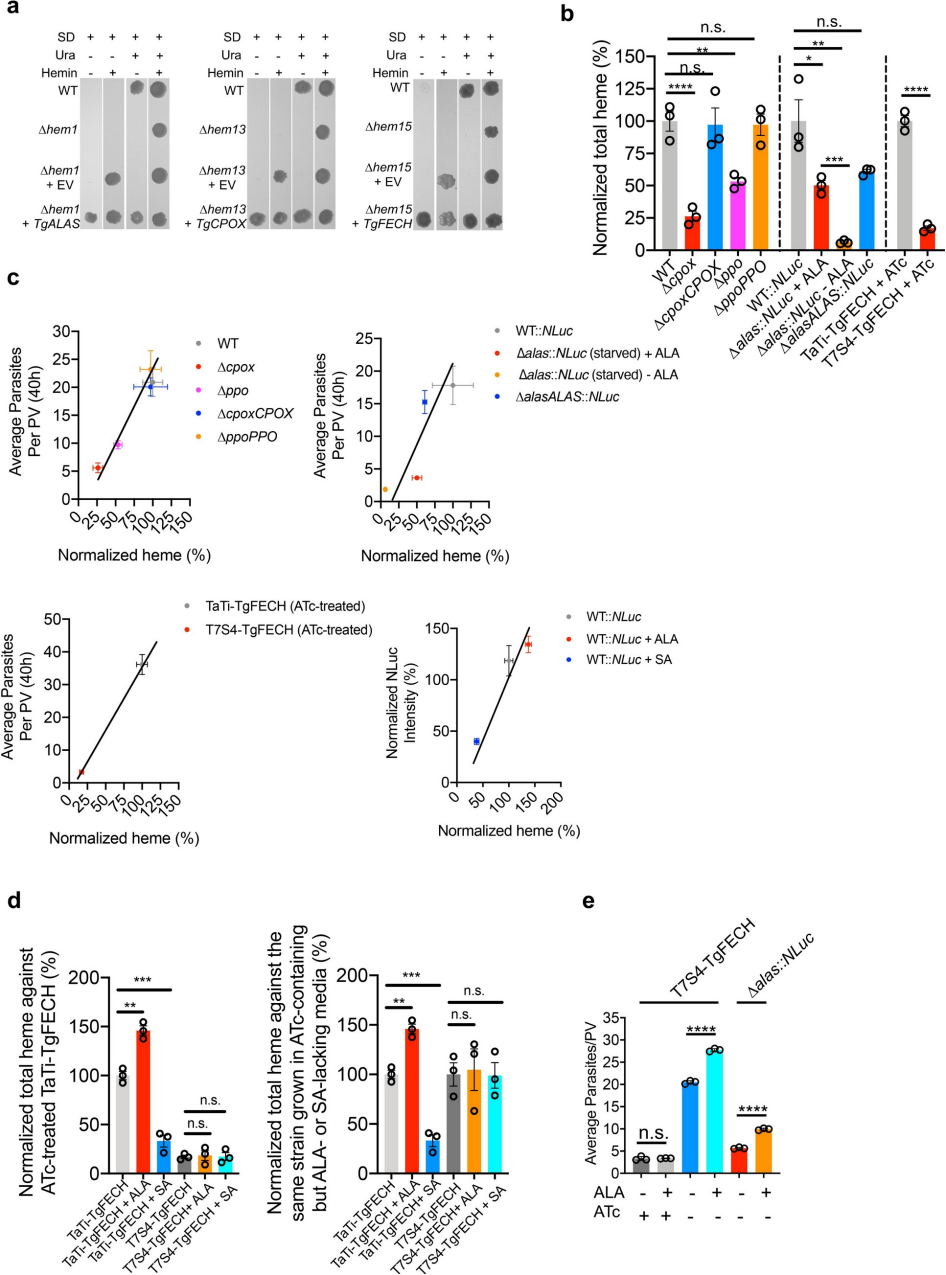
*Toxoplasma* harbors an active *de novo* heme biosynthetic pathway. **a,** Complementation of *Toxoplasma* orthologs of heme biosynthetic genes in the corresponding *S*. *cerevisiae* heme-deficient knockouts. EV, empty vector; SD, synthetic defined medium; Ura, uracil. **b,** Total heme quantification in heme-deficient parasites. A protoporphyrin IX-based fluorescence assay was used to quantify the total heme. The total heme levels in transgenic parasite strains were normalized against the corresponding parental strains. Data represent mean ± SEM of n = 3 biological replicates with 3 technical replicates each. **c,** The average parasite replication or growth rates showed a positive correlation with the normalized heme abundances. Given the different genetic backgrounds of parental strains for individual knockouts, the plots were organized in different groups. The average numbers of parasites per PV for WT, Δ*cpox*, Δ*cpoxCPOX*, Δ*ppo*, and Δ*ppoPPO* were derived from Fig 2E and 2F. The average numbers of parasites per PV for WT::*NLuc*, Δ*alas*::*NLuc* (starved) + ALA, Δ*alas*::*NLuc* (starved)—ALA, and Δ*alasALAS*::*NLuc* were derived from S3A Fig. The average numbers of parasites per PV for TaTi-TgFECH (ATc-treated) and T7S4-TgFECH (ATc-treated) were derived from Fig 2D. The normalized growth rates of WT::*NLuc*, WT::*NLuc* + ALA, and WT::*NLuc* + SA at 48 h post-infection were derived from S7 Fig and S12 Fig. **d,** Chemical interference in heme production in the parasites requires a functional heme biosynthetic pathway in *Toxoplasma*. The *TgFECH* knockdown parasites, along with its parental strain, were treated with ATc for 144 h before a 48-h stimulation or repression of heme production by ALA or SA, respectively. The total heme abundances for each treatment per strain were normalized against the ATc-treated TaTi-TgFECH strain (left panel) or the same strain grown in ATc-containing, but ALA- or SA-lacking, media (right panel). Data represent mean ± SEM from n = 3 biological replicates. **e,** Partial restoration of *TgFECH* expression in ATc-treated T7S4-TgFECH parasites helped them respond to the growth stimulation by ALA treatment. Data represent mean ± SD of n = 3 biological replicates. Statistical significance in all of the studies listed in this figure was calculated by two-tailed unpaired Student’s *t*-test. *, *p*<0.05; **, *p*<0.01; ***, *p*<0.001; ****, *p*<0.0001; n.s., not significant.

To determine that the parasite’s *de novo* heme biosynthesis pathway is active, we complemented *S*. *cerevisiae* heme-deficient mutants lacking *ALAS*, *CPOX*, or *FECH* with the corresponding *Toxoplasma* ortholog genes. The resulting complementation strains had their heme auxotrophy phenotypes restored (Fig 3A), whereas the strains complemented with the empty vector did not grow on heme-free media. Thus, these data establish that TgALAS, TgCPOX, and TgFECH enzymes are functional. Additionally, total heme abundance in the heme biosynthetic gene knockout and knockdown parasites was quantified using a protoporphyrin IX-based fluorescence assay [23]. The total heme abundances in Δ*cpox* and Δ*ppo* were reduced by approximately 75% and 50%, respectively (Fig 3B). The heme level in Δ*alas* was nearly 50% relative to WT parasites when it was grown in medium supplemented with 300 μM ALA, but fell to approximately 10% when ALA was depleted (Fig 3B). Similarly, the heme abundance in the *TgFECH* knockdown strain was decreased to *~*15% compared to its parental strain when the mutant was treated with ATc for 144 h to repress *TgFECH* expression (Fig 3B). Interestingly, the parasite replication or growth rates were positively correlated to the total heme abundance in the parasites (Fig 3C).

We also evaluated whether the *de novo* heme production within *Toxoplasma* is responsive to chemical interference. Succinylacetone (SA), which inhibits *de novo* heme production by targeting TgPBGS activity, or ALA, which stimulates heme production, were used for chemical interrogation of the pathway (Fig 1A). Initially, we determined the half maximal inhibitory concentration (IC_50_) of SA for parasite growth inhibition at 665.5 μM using a luciferase-based growth assay (S7 Fig). Next, we grew T7S4-TgFECH and its parental strain in the ATc-containing media for 144 h, followed by the addition of SA or ALA for an additional 48 h before heme quantification. The heme levels in parental parasites were increased by ~40% with inclusion of ALA and reduced by ~70% due to SA treatment (Fig 3D), compared to DMSO (vehicle control)-treated parasites. However, the incubation of ALA and SA in the ATc-treated T7S4-TgFECH strain did not alter the heme levels in the parasites (Fig 3D). We also found that the inclusion of ALA in the medium improved parasite replication when the *de novo* heme biosynthesis pathway was partially restored in the T7S4-TgFECH strain (Fig 3E). These findings suggest that the parasite’s *de novo* heme biosynthesis pathway actively responds to chemical stimuli for heme production, and the fluctuation in abundance of heme or heme biosynthetic intermediates within host cells does not impact heme levels within the parasites. Taken together, these results indicate that *Toxoplasma* possesses an active heme biosynthesis pathway for its heme supply.

In plants, protoporphyrinogen oxidase (PPO) is involved in heme and chlorophyll production [24]. Chlorophyll is an essential pigment for photosynthesis in plants [25]. When PPO activity is inhibited in plants, the reactant of PPO, protoporphyrinogen IX, leaks into the cytoplasm and is spontaneously oxidized to protoporphyrin IX, which can absorb light to produce highly reactive singlet oxygen that destroys plant cell membranes [26]. Hence, PPO has been widely recognized as a target for herbicide development [26]. A comparison of primary sequences of PPOs from mammals, plants, fungi, protozoans, and bacteria indicates that TgPPO is most closely related to plant orthologs (S8 Fig). Therefore, we evaluated 11 commercial herbicidal PPO inhibitors against WT *T*. *gondii* and identified that the IC_50_ values of 5 compounds ranged from ~130 to 650 μM for the inhibition of parasite growth as determined by a luciferase-based growth assay (S9 Fig).

**Fig 4:**
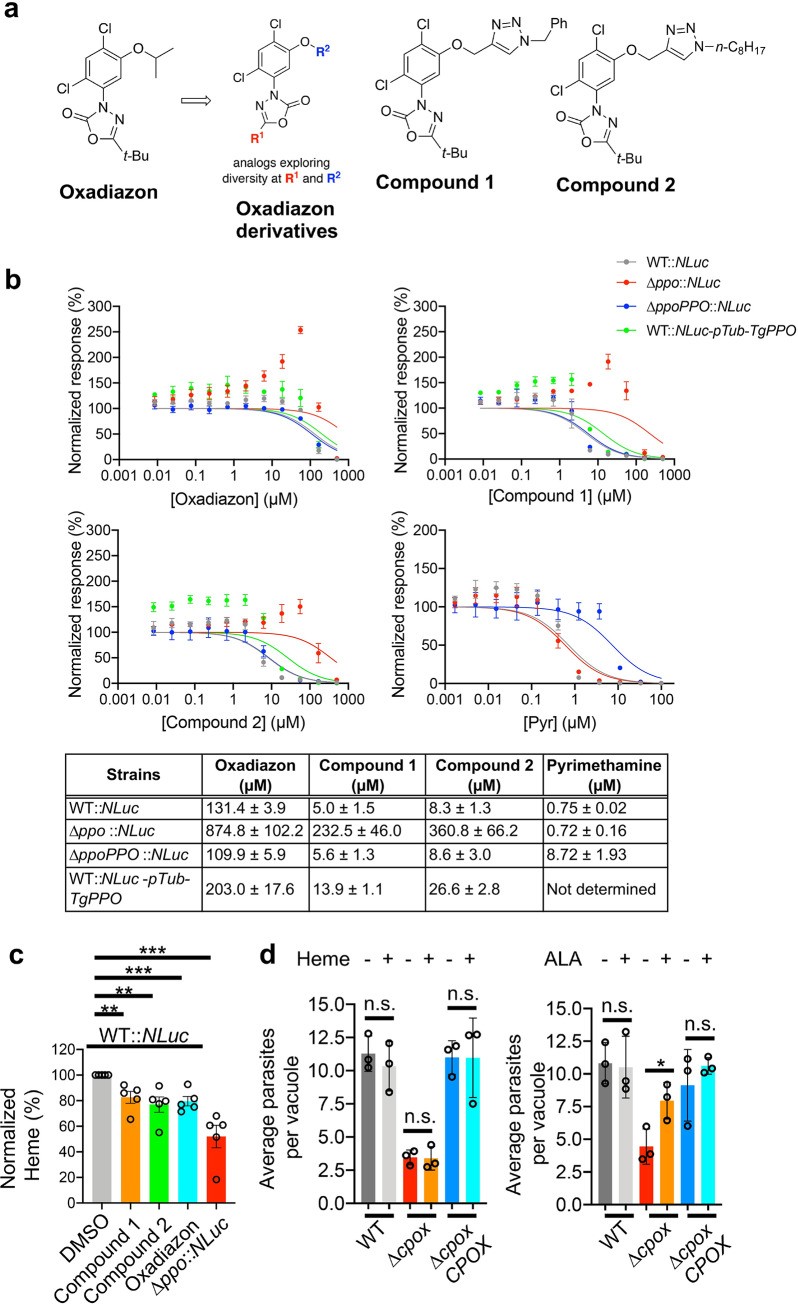
Chemical interrogation of *Toxoplasma’s de novo* heme production by oxadiazon and its derivatives reduced the intracellular growth of the parasites. **a,** Chemical structures of oxadiazon and two derivatives. **b,** Efficacy determination of oxadiazon and its derivatives in the inhibition of WT *Toxoplasma* growth using a luciferase-based growth assay. The Δ*ppo*::*NLuc*, Δ*ppoPPO*::*NLuc*, and WT::*NLuc-pTub-TgPPO* strains were included for evaluating the target specificity of the inhibitors to TgPPO. Pyrimethamine, an antibiotic targeting folic acid metabolism that is irrelevant to heme biosynthesis, was also included for assessing the specificity of these PPO inhibitors. Data shown in the table represent mean ± SEM of n = 3 biological replicates with 3 technical replicates each. The standard errors for individual IC_50_s listed in the table were calculated from the IC_50_s derived from 3 independent biological replicates for each inhibitor. The IC_50_ values were obtained by curve fitting using the function of “normalized response” vs. “[inhibitor]”, under the “dose-response-inhibition” regression program embedded in GraphPad Prism software (8^th^ version). **c,** Heme levels were reduced in the parasites upon treatment with oxadiazon and its derivatives. Data shown here represent mean ± SEM of n = 5 biological replicates. **d,** *Toxoplasma* was incapable of taking up extracellular heme to support its intracellular growth. Data represent mean ± SD of n = 3 biological replicates. Statistical significance for the assays described in this figure was determined by two-tailed unpaired Student’s *t*-test. *, *p*<0.05; **, *p*<0.01; ***, *p*<0.001; n.s., not significant.

Because oxadiazon was the most potent compound identified, and in light of its potential for additional medicinal chemistry optimization, we next evaluated two derivatives of the scaffold by modifying a structural homolog, oxadiargyl, by means of straightforward cycloaddition chemistry (Fig 4A). Both derivatives had improved potency, with IC_50_ values approximately 15-25-fold lower than oxadiazon (Fig 4B). In addition, we found that the Δ*ppo* parasites were less sensitive to these compounds than either WT or Δ*ppoPPO* strains (Fig 4B). To further validate that TgPPO is the *in vivo* target recognized by oxadiazon and its derivatives, we expressed *TgPPO* under the *Toxoplasma* tubulin promoter in WT::*NLuc* parental parasites to create a *TgPPO* overexpression strain, named WT::*NLuc-pTub-TgPPO*. By quantitative PCR, the transcript level of *TgPPO* in WT::*NLuc-pTub-TgPPO* was increased by ~12-fold compared to that of the parental strain (S10 Fig). Upon overexpression of *TgPPO*, the IC_50_ for WT::*NLuc-pTub-TgPPO* were increased by 1.5 to 3.2-fold relative to WT::*NLuc* strain (Fig 4B). These additional findings suggest that these compounds were targeting TgPPO. Interestingly, we noticed that the oxadiazon and its derivatives all enhanced the intracellular growth of Δ*ppo* parasites when they were supplemented in the media in the range from 2.1 to 55.5 μM, showing the maximal growth increase at 55.5 μM for all three PPO inhibitors (Fig 4B). A future follow-up study will be performed to explore the molecular mechanisms underlying this interesting phenomenon. The differences in growth were not noted when WT or Δ*ppo* parasites were treated with pyrimethamine, a clinical antibiotic prescribed against acute toxoplasmosis by inhibiting folic acid synthesis (Fig 4B). The Δ*ppoPPO* strain was less sensitive to pyrimethamine treatment because a pyrimethamine resistance cassette had been used to generate the *TgPPO* complementation plasmid. To test whether these PPO inhibitors reduced heme production in the parasites, the parental WT::*NLuc* parasites were treated with 200 μM oxadiazon, or 5 μM Compound 1, or 10 μM Compound 2 for 4 days prior to heme quantification. Our findings revealed that the heme abundance in WT parasites upon treatment with oxadiazon or its derivatives was reduced by approximately 15–20% relative to the DMSO-treated parental strain (Fig 4C), further supporting the notion that these chemicals target the heme biosynthesis pathway in the parasites. Notably, none of the compounds were toxic to human foreskin fibroblasts based on an Alarmar Blue-derived cell viability assay (S11 Fig).

Given the unsuccessful attempts to delete *TgFECH* in the medium supplemented with heme, we speculate that *Toxoplasma* is unable to acquire adequate heme from the host to support its intracellular growth due to the lack of heme transporter. Supporting this, homology searches using several identified heme transporters as templates [27,28] failed to identify candidate heme transporters in the *Toxoplasma* genome (ToxoDB database). We also used the Δ*cpox* cell line as a proxy to test whether *Toxoplasma* can incorporate extracellular heme to support intracellular replication. There was no significant difference in the quantification of Δ*cpox* replication between standard growth medium and the medium supplemented with 10 μM heme (Fig 4D). A Δ*cpox* replication comparison between standard growth medium and the medium containing 300 μM ALA was included as a positive control (Fig 4D) since *Toxoplasma* can incorporate extracellular ALA into its heme biosynthesis pathway as described in a previous publication [6]. A similar observation was seen in the intracellular growth of Δ*cpox* parasites when comparing growth within heme-depleted and heme-enriched media (S12 Fig). These observations supported the concept that the development of a strategy for specific chemical interrogation of the parasite’s heme biosynthesis shows the therapeutic potential for controlling toxoplasmosis.

## Concluding remarks

In conclusion, we revealed that *Toxoplasma* parasites harbor an active plant-like heme biosynthesis pathway and principally rely on this pathway to produce heme for their intracellular growth and acute virulence. In addition, the current antibiotics against *Toxoplasma* trigger strong side effects in some groups of patients and have limited efficacy on congenital toxoplasmosis. Thus, an urgent need for new therapeutics exists. Our findings shed light on the identification of potential novel therapeutic targets within the parasite’s heme biosynthesis pathway for the clinical management of *Toxoplasma* infections.

## Materials and methods

### Ethics statement

This study was conducted in compliance with the Public Health Service Policy on Humane Care and Use of Laboratory Animals and Association for the Assessment and Accreditation of Laboratory Animal Care guidelines. Mice that appeared moribund were humanely euthanized via CO_2_ overdose, in compliance with the protocol approved by Clemson University’s Institutional Animal Care and Use Committee (Animal Welfare Assurance A3737-01, protocol number AUP2016-012). This method of euthanasia is consistent with the recommendations of the Panel on Euthanasia of the American Veterinary Medical Association.

### Chemicals and reagents

5-aminolevulinic acid hydrochloride (ALA) was purchased from Ark Pharm (catalog number: AK-30504). Succinylacetone (SA) was obtained from Sigma-Aldrich (catalog number: D1415-100MG). Anhydrotetracycline hydrochloride (ATc) was acquired from Cayman Chemical (catalog number: 10009542). The herbicidal PPO inhibitors were ordered from Chem Service. Other chemicals used in this work were analytical grade and were purchased from VWR unless otherwise indicated. All oligonucleotide primers listed in S4 Table for this study were purchased from Eurofins.

### Host cells and parasite culture

Human foreskin fibroblasts (HFFs) were obtained from the American Type Culture Collection (ATCC, catalog number: SCRC-1041). Tissue cultures were maintained at 37°C with 5% CO_2_ in D10 medium (Dulbecco’s Modified Eagle Medium, 4.5 g/L glucose, VWR) supplemented with 10% Cosmic Calf serum (HycloneTM, GE Healthcare Life Sciences SH30087.03), 10 mM HEPES, additional 2 mM L-glutamine, and 10 mM Pen/Strep as instructed by the vendor’s manual. The HFF cells were tested for mycoplasma contamination every month. *Toxoplasma* strain RHΔ*ku80*Δ*hxg* and TaTiΔ*ku80*Δ*hxg* were obtained from the Carruthers Lab (Univ of Michigan) and the Striepen Lab (Univ of Pennsylvania), respectively, who originally created these *Toxoplasma* parental strains [29,30]. All *Toxoplasma* strains were maintained *in vitro* by serial passage on HFFs. The Δ*alas*::*NLuc* strain was kept in the D10 medium supplemented with 300 μM ALA.

### Generation of transgenic *T*. *gondii* strains (S2 Table)

#### (1) Endogenously epitope-tagging the heme biosynthetic genes

The RHΔ*ku80*Δ*hxg* strain was used as the parental strain for endogenous gene tagging. The *TgALAS*, *TgALAD*, *TgCPOX*, *TgPPO*, and *TgFECH* were endogenously tagged with a 3xHA epitope at their C-termini by CRISPR-Cas9-mediated double crossover recombination as described previously [31]. The *TgPBGD*, *TgUROS*, and *TgUROD* were endogenously tagged with a 3xmyc tag C-terminally using similar methods as mentioned above. Briefly, the guide RNA selected to target the 3’-end of the coding sequence for individual genes was incorporated into a guide RNA expression construct as described previously [32]. This construct also encodes Cas9 protein that assists the guide RNA in generating a double-stranded break at the end of genes of interest. Fifty base pairs of homologous regions upstream and downstream stop codon were engineered into forward and reverse primers, respectively. By PCR, they were flanked at the 5’- and 3’-ends of the epitope tag and a drug resistance cassette, respectively, to create a repair template. The guide RNA/Cas9 expression plasmids and the repair template were co-introduced into the parental strain by electroporation. Through double-crossover homologous recombination, the epitope tag and drug resistance cassette were incorporated after the last codon of individual genes (S1A Fig). The resulting strains expressing epitope-tagged genes were drug selected and cloned out. The primers used for the epitope tagging of individual genes are listed in the S4 Table.

#### (2) Genetic ablation of the heme biosynthetic genes

A similar CRISPR-Cas9-based gene editing strategy was conducted for the deletion of *TgCPOX* and *TgPPO* in the RHΔ*ku80*Δ*hxg* strain, and the ablation of *TgALAS* in the RHΔ*ku80*::*NLuc* strain generated in our previous study [31]. Similarly, the 50-bp homologous upstream and downstream DNA sequences of the start and stop codons of the corresponding gene, respectively, were incorporated into primers for amplification of the drug resistance cassette by PCR. In the final PCR product, the homologous regions located at both ends of the target gene facilitated the replacement of the gene of interest with a drug resistance cassette by double crossover recombination. For individual genes, the PCR product was mixed with the corresponding guide RNA used for the endogenous gene tagging and electroporated into RHΔ*ku80*Δ*hxg* or RHΔ*ku80Δhxg*::*NLuc* strains for knockout mutant generation. The correct clones were drug selected and cloned out as described previously [31]. PCR was used to verify the integration of the drug resistance cassette in the correct locus (S2A and S2B Fig). The primers used for the deletion of individual genes and the verification of the knockout mutants are listed in the S4 Table and S2 Text. Please refer to S1 Text for more details.

#### (3) Complementation of the heme-deficient *Toxoplasma* knockouts

To restore the gene expression in the corresponding heme-deficient knockouts, we PCR-amplified the coding sequences of the individual heme biosynthetic genes from the *Toxoplasma* cDNA library and the corresponding 1kb 5’- and 3’-UTR (untranslated regions) from the *Toxoplasma* genomic DNA. All three PCR products for individual genes were assembled into a plasmid vector carrying the pyrimethamine resistance cassette to create the corresponding complementation constructs. For both *TgPPO* and *TgALAS* complementation constructs, a 1kb DNA fragment localized to ~ 6kb upstream *TgKU80* gene was included in the complementation construct to facilitate a single insertion of the complemented gene into the genome of the knockout strain to keep its expression similar to its endogenous expression level. Approximately twenty micrograms of complementation plasmids were used for transfection. After stabilization by drug selection, the complemented parasites were cloned out. PCR was used to verify the integration of the exogenous genes introduced into the parasite’s genome. Additionally, the total RNA from the knockouts, their parental strains, and the corresponding complementation strains were purified and tested by RT-PCR to confirm the loss of their messenger RNAs in the knockouts and the restored expression in the complementation strains (S2C Fig). The primers used for the complementation of individual genes in the corresponding *Toxoplasma* knockout mutants and the verification of their integration are listed in the S4 Table.

#### (4) Generation of the ferrochelatase (TgFECH) knockdown strain using the tetracycline-controlled TET-OFF system

The TaTiΔ*ku80*Δ*hxg* strain carries the required genetic elements to suppress gene expression in response to the treatment of anhydrotetracycline (ATc) [30] and therefore is being used as a parental strain to knock down the *TgFECH* gene. To help recognize and evaluate the expression repression of *TgFECH*, we first endogenously tagged it with a 3xmyc epitope C-terminally, as described above, to create a TaTiΔ*ku80*::*TgFECH-3xmyc* strain. A 1kb region upstream the start codon of *TgFECH* was recognized as the promoter region and targeted for replacement with a tetracycline-responsive T7S4 promoter. To achieve this, the 50-bp regions upstream and downstream the *TgFECH* start codon were incorporated at the 5’- and 3’-ends of the pyrimethamine resistance cassette and a tetracycline-responsive T7S4 promoter by PCR using a similar strategy as mentioned above. A single clone of TaTiΔ*ku80*::*TgFECH-3xmyc* was transfected with the T7S4 promoter-encoding PCR product, along with a guide RNA expression construct recognizing the 5’-end of *TgFECH* gene, to create a T7S4-TgFECH-3xmyc strain. Please refer to S4A Fig for the schematic description of the detailed steps. The epitope tag integration and the promoter replacement were confirmed by PCR (S4B Fig). The primers used for the generation of the *TgFECH* knockdown strain and its verification are listed in the S4 Table.

#### (5) Generation of nanoLuc luciferase (NLuc)-expressing parasite strains

The *NLuc* expression plasmids were introduced into individual Δ*cpox* and Δ*ppo* parasite strains and the corresponding Δ*cpoxCPOX* and Δ*ppoPPO* complementation strains. The resulting *NLuc*-expressing strains were drug-selected and cloned out. Individual clones for each strain were screened by their luciferase activities to help select clones expressing similar levels of NLuc activity. These clones were maintained for downstream assays including intracellular growth and IC_50_ determination for PPO inhibitors.

#### (6) Generation of a transgenic strain overexpressing *TgPPO*

The coding sequence of *TgPPO* was PCR-amplified and placed downstream a *Toxoplasma* tubulin promoter for its overexpression in *Toxoplasma* parasites. The resulting plasmid also encodes a pyrimethamine resistance cassette for drug-selection of the parasites that overexpress *TgPPO*. The WT::*NLuc* parasites were transfected with the *TgPPO* overexpression plasmid using the standard electroporation protocol documented in our previous publication [31]. The transfectants were further stabilized by drug selection and cloned out. PCR was used to screen the clones carrying the exogenously introduced plasmid. Quantitative PCR was also performed on the screened clones to evaluate the level of *TgPPO* transcript for confirmation of its overexpression before drug efficacy testing.

### Quantitative PCR (qPCR) assay

*Toxoplasma* parasites were grown in HFF cells for 2 days before total RNA purification. Filter-purified parasites were subjected to a trizol-based method for total RNA extraction by using the Direct-zol RNA MiniPrep Plus kit (Zymo). Approximately 300 ng of total RNA was used to detect and/or quantify the steady levels of transcripts for individual genes by using the Luna Universal One-Step RT-PCR kit (NEB). The data acquisition for the qPCR assay was performed using the BioRad CFX96 Touch Real-Time PCR detection system. The double delta cycle threshold (ΔΔCT) analysis was used to calculate the relative abundances of the transcripts for individual genes in transgenic parasite strains compared to that in WT parasites using the Bio-Rad CFX Maestro software. *TgActin* was used for normalization as the housekeeping gene.

### SDS-PAGE and immunoblotting

*Toxoplasma* parasites were grown and maintained in HFFs for a routine 2-day pass. Filter-purified parasites were resuspended in 1x SDS-PAGE sample buffer (40 mM Tris, pH 6.8, 1% SDS, 5% glycerol, 0.0003% bromophenol blue, 50 mM DTT) and boiled for 10 min prior to resolving by SDS-PAGE. Separated polypeptides were transferred to PVDF membranes via semi-dry protein transfer. For chemiluminescence-based detection, blots were blocked with 5% non-fat milk, incubated with primary antibody diluted in 1% non-fat milk in PBS, followed by probing with goat anti-mouse or anti-rabbit IgG antibodies conjugated with horseradish peroxidase as the secondary antibody diluted in 1% non-fat milk in PBS. Immunoblots were developed with SuperSignal WestPico chemiluminescent substrate (Thermo Fisher). For fluorescence-based detection, blots were blocked with 1.25% fish gelatin in 50 mM Tris, pH 7.4, 0.15 M NaCl, incubated with primary antibody diluted in wash buffer (0.1% Tween-20 in PBS), followed by probing with goat anti-mouse or anti-rabbit IgG antibodies conjugated with 680RD or 800CW dyes as the secondary antibody (LI-COR). The chemiluminescence and fluorescence signals were captured using the Azure C600 Imaging System.

### Immunofluorescence microscopy

Freshly lysed parasites were used to infect confluent HFF cells pre-seeded in an 8-well chamber slide for 1 h (pulse-invaded parasites) or 28–40 h (replicated parasites). Immunofluorescence was performed as described previously [31]. Images were viewed and digitally captured using a Leica CCD camera equipped with a Leica DMi8 inverted epifluorescence microscope and processed with Leica LAS X software.

### PPIX-based total heme quantification

The parasites were grown in HFFs for 2 days in regular D10 medium or the medium supplemented with chemicals indicated in the text before harvest. Freshly lysed parasites were syringed, filter-purified, and washed in ice-cold PBS buffer. The parasites were finally resuspended in 400 μl of ice-cold PBS buffer, and sonicated on ice using a Branson analog sonifier with a mini horn at the output intensity setting of 3 and duty% of 20%, at a 10-sec interval, for 4 times. A thirty-second rest was set between each sonication to avoid overheating. One hundred microliters of samples were mixed with 900 μl of 2 M oxalic acid in solid black Eppendorf tubes and boiled for 30 min. Similarly, each sample was duplicated in the same way without boiling, serving as a background reading. The heme solutions at 0, 5, 14.7, 44.3, 133, 400, and 1200 nM were included in the assay to produce a standard curve. The fluorescence was recorded at 400 nm excitation wavelength and 608 nm emission wavelength using the BioTek H1 Hybrid plate reader. The fluorescence of non-boiled samples will be subtracted from that of boiled samples. The final fluorescence was normalized against the amount of parasites for comparison. The normalized heme abundance in WT parasites was set at 100% to calculate the relative heme abundance in other strains.

### Plaque assay of *T*. *gondii*

Freshly egressed parasites were filter-purified and resuspended in D10 medium. Two hundred parasites were inoculated into a 6-well plate seeded with confluent HFFs. The plate was incubated at 37°C with 5% CO_2_ for 7 days without disturbance. The developed plaques were stained with 0.002% crystal violet in 70% ethanol for 5 min and washed with water to enhance visualization of developed plaques. The plaques were scanned to capture the images of entire wells or observed under 25x magnification using a Leica DMi8 microscope. At least 50 individual plaques were photographed for measurement of their sizes. The plaque size was plotted for comparison.

### Replication assay of *T*. *gondii*

For the parasite replication assay, freshly lysed parasites were filter-purified and inoculated into individual wells of an 8-well chamber slide pre-seeded with HFF cells at approximately 1 x 10^5^ cells per well and incubated at 37°C with 5% CO_2_. Non-invaded parasites were washed off at 4 h post-infection. Invaded parasites were allowed to infect host cells for an additional 24 and 36 h before fixation. The infected host cells were stained with monoclonal anti-TgGRA7 (1:2,000) antibody and DAPI to help distinguish individual parasitophorous vacuoles (PVs) and parasite nuclei, respectively. Slides were subjected to standard immunofluorescence microscopy for imaging. One hundred PVs were enumerated for each strain and plotted as average parasites per PV for comparison.

### Bioluminescence-based growth assay

Individual strains were purified, resuspended in D10 medium, and inoculated into 96-well plate pre-seeded with confluent HFFs. Parasites were allowed to invade host cells for 4 h at 37°C with 5% CO_2_ before washing away non-invaded parasites, and the bioluminescence of invaded parasites at 4 h post-infection was measured for normalization. Invaded parasites were incubated at 37°C with 5% CO_2_ for an additional 96 h, and their bioluminescence was measured every 24 h and normalized against the signal derived at 4 h for the calculation of the fold change of bioluminescence, which reflects parasite intracellular growth rate.

### Mouse studies

Six- to eight-week-old, outbred CD-1 mice were infected with WT or transgenic parasite strains diluted in PBS, with inocula indicated in the text, by subcutaneous injection. The infected mice were monitored daily for developing symptoms over 30 days. CO_2_ overdose was used to euthanize mice. The seroconversion of the surviving mice was tested by enzyme-linked immunosorbent assay (ELISA). The surviving mice were allowed to rest for 10 days, prior to a subcutaneous challenge injection with 1,000 WT parasites, and were kept for daily monitoring of survival for an additional 30 days. The mice survival curve was plotted for statistical significance calculation by using the Log-rank (Mantel-Cox) test.

### Yeast complementation assay

To test the functionality of the *Toxoplasma* orthologs of the heme biosynthetic proteins, we complemented these orthologs in the corresponding yeast knockouts (S3 Table). First, we PCR-amplified the G418 resistance cassette from the plasmid pFA6a-6xGLY-Myc-kanMX6 (Addgene, plasmid #:20769), flanked by 50-bp upstream and downstream regions of the start and stop codons of the corresponding heme biosynthetic gene, respectively. Approximately 5 μg of PCR products were transformed into the haploid yeast strain BY4741 (GE Healthcare Dharmacon Inc., catalog number: YSC 1048) to remove the coding sequences of heme biosynthetic genes by homologous recombination using standard yeast transformation procedures (S6A Fig). The transformants were selected on YPD plates containing 200 μg/ml of G418 and 15 μg/ml hemin. After two to three days of incubation, correct yeast knockouts were identified by PCR (S6B Fig). Next, we amplified the coding sequences of the corresponding *Toxoplasma* orthologs of individual heme biosynthetic genes from the *Toxoplasma* cDNA library by PCR and drove its expression under the promoter of the yeast TEF1 gene in the pXP318 yeast expression construct to create a complementation construct. The yeast complementation construct was chemically introduced into the corresponding yeast knockout. The pXP318 encodes a uracil biosynthesis gene, while the WT yeast strain shows a uracil auxotrophy phenotype. Therefore, the successful transformants receiving pXP318 or pXP318-derived plasmids did not require the addition of uracil in the growth medium. The yeast knockouts complemented with the corresponding *Toxoplasma* orthologs were also patched onto plates lacking exogenously added heme to evaluate a heme autotrophy phenotype. The primers used for the yeast knockout generation and complementation of individual *Toxoplasma* orthologs are listed in the S4 Table and S2 Text.

### Synthesis of oxadiazon derivatives

All reagents were purchased from commercial sources and used without purification. ^1^H and ^13^C NMR spectra were collected on Bruker 300 MHz NMR spectrometers using CDCl_3_ as the solvent. Chemical shifts are reported in parts per million (ppm). Spectra are referenced to residual solvent peaks. Infrared spectroscopy data were collected using an IR Affinity-1S instrument (with MIRacle 10 single reflection ATR accessory). Flash silica gel (40–63 μm) was used for column chromatography. The compounds were characterized by ^1^H and ^13^C NMR, ATR-FTIR, and HRMS. HRMS data were collected using an instrument equipped with electrospray ionization in positive mode (ESI+) and a Time Of Flight (TOF) detector (S13 and S14 Figs).

General Procedure for Oxadiargyl-triazole analogs [33]: Oxadiargyl (50 mg, 0.145 mmol, 1.0 equiv) was dissolved in DCM/H_2_O, 1:1 (0.5 mL) in a 20 mL round-bottomed flask equipped with a stir bar. The appropriate azide (0.175 mmol, 1.2 equiv) was added, followed by anhydrous CuSO_4_ (1 mg, 0.007 mmol, 0.05 equiv) and sodium ascorbate (4 mg, 0.02 mmol, 0.15 equiv). The resulting solution was then stirred vigorously at room temperature for 4 h. The reaction was diluted with DCM (5 mL) and water (5 mL). The organic layer was washed with saturated aq. brine, dried over anhydrous sodium sulfate, and concentrated by rotary evaporation to provide the crude product as a brown oil. The isolate was purified by silica gel flash column chromatography under the following gradient: hexanes (20 mL), ethyl acetate/hexanes (95:5; 20 mL), ethyl acetate/hexanes (90:10; 50 mL), ethyl acetate/hexanes (80:20; 50 mL), ethyl acetate/hexanes (70:30; 50 mL), ethyl acetate/hexanes (50:50; 50 mL) to afford an opaque oil (62–87% isolated yield).

#### 3-(5-((1-benzyl-*1H*-1,2,3-triazol-4-yl)methoxy)-2,4-dichlorophenyl)-5-(*tert*-butyl)-1,3,4-oxadiazol-2(*3H*)-one (Compound 1)

Compound 1 was obtained in 62% yield using benzyl azide [34] (23 mg, 0.18 mmol, 1.2 equiv) as the azide reagent.

Opaque oil; Yield: 62% (69 mg); R_*f*_ 0.54 (1:1 ethyl acetate/hexanes, UV); IR: (film) = 2970, 2924, 2856, 2094, 1782, 1489, 1246, 752, 717 cm^-1^; ^1^H NMR: (300 MHz, CDCl_3_) δ 1.39 (s, 9H), δ 5.29 (s, 2H), δ 5.55 (s, 2H), δ 7.24 (s, 1H), δ 7.30 (m, 2H), δ = 7.38 (m, 2H), δ 7.52 (s, 1H), δ 7.61 (s, 1H); ^13^C{^1^H} NMR: (75 MHz, CDCl_3_) δ = 27.0, 32.9, 54.3, 63.7, 113.8, 123.0, 123.9, 125.2, 128.1, 128.9, 129.2, 131.4, 134.3, 142.6, 152.0, 152.9, 163.6; HRMS (ESI-TOF): Calcd. for C_22_H_22_Cl_2_N_5_O_3_, [M+H]^+^ 474.1100 found m/z 474.1099.

#### 5-(*tert*-butyl)-3-(2,4-dichloro-5-((1-octyl-*1H*-1,2,3-triazol-4-yl)methoxy)phenyl)-1,3,4-oxadiazol-2(*3H*)-one (Compound 2)

Compound 2 was obtained in 87% yield using octyl azide [35] (24 mg, 0.18 mmol, 1.2 equiv) as the azide reagent.

Opaque oil; Yield 87% (72 mg); R_*f*_ 0.50 (1:1 ethyl acetate/hexanes, UV); IR: (film) = 2951, 2924, 2854, 1786, 1479, 1246, 1126, 1041, 733 cm^-1^; ^1^H NMR: (300 MHz, CDCl_3_) δ = 0.87 (t, 3H, *J* = 7 Hz), 1.26 (m, 12H), 1.38 (s, 9H), 1.92 (p, 2H, *J* = 7 Hz), 4.36 (t, 2H, *J* = 7 Hz), 7.26 (s, 1H), 7.52 (s, 1H), 7.68 (s, 1H); ^13^C{^1^H} NMR: (75 MHz, CDCl_3_) δ = 27.0, 32.9, 54.3, 63.7, 113.8, 122.9, 123.9, 125.1, 131.4, 142.6, 152.0, 152.9, 163.6; HRMS (ESI+-TOF): Calcd for C_23_H_31_Cl_2_N_5_O_3_, [M+H]^+^ 496.1882 Found m/z 496.1878.

### *In vitro* measurement of IC_50_s for chemical inhibitors

General procedures: Individual parasite strains expressing NanoLuc luciferase were used to infect confluent HFFs pre-seeded into a 96-well solid white plate with inocula of 1,000 tachyzoites per well for WT::*NLuc* and Δ*ppoPPO*::*NLuc* strains, and 3,000 tachyzoites per well for the Δ*ppo*::*NLuc* strain. A higher inoculum of Δ*ppo*::*NLuc* was adjusted in the assay to help accurately quantify fold change of luciferase activity due to its slow growth. Purified parasites were resuspended in phenol red-free D10 medium, and 150 μl of parasite resuspension for individual strains were inoculated in each well. Parasites were initially incubated at 37°C with 5% CO_2_ for 4 h to facilitate host invasion. Next, the media were gently aspirated and replaced with the phenol red-free D10 media containing individual chemical inhibitors at the serially diluted concentrations listed below. Control medium without an inhibitor was included for luciferase activity normalization. After a 2- to 4-day incubation with chemical inhibitors, the media were gently aspirated to avoid disturbance of the infected HFF monolayer, replaced with 12.5 μM Coelenterazine h in lysis buffer (100 mM 4-Morpholineethanesulfonic acid (MES) pH 6.0, 1 mM trans-1,2-Diaminocyclohexane-N,N,N′,N′-tetraacetic acid (CDTA), 0.5% (v/v) Tergitol, 0.05% (v/v) Mazu DF 204, 150 mM KCl, 1 mM DTT, and 35 mM thiourea) [36], and incubated at room temperature for 10 min to fully lyse cells prior to luciferase activity quantification. The signals from wells of each individual concentration of inhibitors were recorded using a BioTek plate reader and normalized against the luciferase activities derived from the infected cells incubated in plain medium. The normalized luciferase activities were plotted against the concentrations of inhibitors to calculate the IC_50_ values for individual inhibitors using the built-in “Dose-response-inhibition” program in GraphPad Prism software (8^th^ version).

#### Measurement of IC_50_s for the PPO inhibitors

The serial concentrations of PPO inhibitors were 500.0, 125.0, 31.3, 7.81, 1.95, 0.488, 0.122, 3.05x10^-2^, and 7.63x10^-3^ μM. The efficacies of these inhibitors were only tested on WT::*NLuc* parasites after 4 days of growth.

#### Measurement of IC_50_s for oxadiazon and its derivatives

The serial concentrations of oxadiazon and its derivatives were 500.0, 166.7, 55.6, 18.5, 6.17, 2.06, 6.8x10^-1^, 2.3x10^-1^, 7.6x10^-2^, 2.5x10^-2^, and 8.5x10^-3^ μM. The efficacies of these inhibitors were tested on WT::*NLuc*, Δ*ppo*::*NLuc*, and Δ*ppoPPO*::*NLuc* parasites after 4 days of growth.

#### Measurement of IC_50_ for pyrimethamine

The serial concentrations of pyrimethamine included in the media were 100.0, 33.3. 11.1, 3.70, 1.23, 0.41, 0.14. 0.045, 0.015, 5.1x10^-3^, and 1.7x10^-3^ μM. The efficacy of pyrimethamine was tested on WT::*NLuc*, Δ*ppo*::*NLuc*, and Δ*ppoPPO*::*NLuc* parasites after 4 days of growth.

#### Measurement of IC_50_ for succinylacetone

The serial concentrations of succinylacetone included in the media were 6,666.6, 2,222.2, 740.7, 246.9, 82.3, 27.4, 9.14, 3.05, and 1.02 μM. The IC_50_ value of succinylacetone was determined using its inhibition on 2-day growth of WT::*NLuc* parasites.

### Resazurin-based cell viability assay

The HFFs were seeded in 96-well plates and grown in regular D10 medium. After the host cells became confluent, they were incubated in media containing the tested PPO inhibitors at the serially diluted concentrations of 500.0, 166.7, 55.6, 18.5, 6.17, 2.06, 6.8x10^-1^, 2.3x10^-1^, 7.6x10^-2^, 2.5x10^-2^, and 8.5x10^-3^ μM, or the media containing Triton X-100 at 1070.0, 356.7, 118.9, 39.6, 13.2, 4.40, 1.47, 0.49, 0.16, 0.054, and 0.018 μg/ml for 4 days. The host cells grown in regular D10 medium were included as the control for normalization. The resazurin was diluted at 0.004% in regular D10 medium and incubated with host cells at 37°C with 5% CO_2_ for 4 h before absorbance measurement at 570 and 600 nm using a BioTek H1 Hybrid plate reader. A resazurin-containing medium control that was not incubated with host cells was included to calculate the absorbance ratio of oxidized resazurin at 570 nm vs. 600 nm, termed OD(blank)_570/600_. The normalized cell viability was calculated using the following equation: [OD(inhibitor)_570_—OD(inhibitor)_600_ x OD(blank)_570/600_]/ [OD(medium)_570_—OD(medium)_600_ x OD(blank)_570/600_] *100%.

### Phylogenetic tree construction

Polypeptide sequences of 17 PPO orthologs from animals, plants, bacteria, protozoa, and fungi were retrieved from the Uniprot database (www.uniprot.org). The sequences were aligned using the CLUSTAL MUSCLE (MUltiple Sequence Comparison by Log-Expectation) alignment tools [37,38]. The resulting sequence alignment was used to construct a phylogenetic tree by using the Neighbor-Joining tree analysis [39] built into the Geneious software. Bootstrap values based on 10,000 replicates are shown. The accession IDs used for MUSCLE alignment were listed in S8 Fig.

### Statistical significance calculation

The statistical significance calculation in this study was performed using GraphPad Prism software (8^th^ version). The methods for individual assays were indicated in the figure captions.

## Supporting information

S1 Fig Endogenous epitope tagging of the *Toxoplasma* heme biosynthetic genes. **a,** Schematic illustration of the endogenous gene epitope tagging in *Toxoplasma*. In brief, a 3xHA or 3xmyc tag was fused at the C-termini of the genes of interest using a CRISPR-Cas9-based cloning strategy. **b,** TgUROD was localized to the apicoplast, instead of the cytoplasm. TgActin was used as a cytoplasm marker. Bar = 2 μm. **c,** Immunoblotting analysis was used to confirm the expression of the epitope-tagged genes. The bands labeled with asterisks were derived from non-specific binding to antibodies. For TgALAS-3xHA, two protein fragments denoted by the filled arrowheads, migrating at ~80 kDa and ~40 kDa, represent the full-length and truncated TgALAS proteins, respectively. The *TgPBGS* gene only can be endogenously epitope-tagged in a transient manner. The protein lysate was purified from parasites that lysed immediately after transfection with the guide RNA expression construct and a TgPBGS-3xHA tagging DNA fragment. The guide RNA expression construct also expressed the 3xHA-tagged Cas9 proteins. Based on the predicted molecular weight, the band denoted by an unfilled arrowhead was derived from 3xHA-tagged Cas9. GOI, gene of interest; DRC, drug resistance cassette.
(TIF)

S2 Fig Genetic deletion of the *Toxoplasma* heme biosynthetic genes. **a,** Schematic illustration of a general CRISPR-Cas9-based strategy for gene deletion in *Toxoplasma*. **b,** PCR confirmation of gene ablation. The genomic locations of the primers used in PCR amplification were indicated in the scheme. **c,** The loss of messenger RNA of the genes of interest was confirmed by reverse-transcription PCR (RT-PCR). **d,** The correct integration of the drug resistance cassette into the *TgFECH* locus during gene deletion was detected by PCR. However, the knockout parasites cannot be cloned probably due to its non-viability. GOI, gene of interest; DRC, drug resistance cassette.
(TIF)

S3 Fig *TgALAS*-deletion mutant showed defective replication and plaque formation and required extracellular ALA for intracellular growth. **a,** Replication comparison of the ALA-starved and non-starved Δ*alas*::*NLuc* parasites in the media containing or lacking ALA. The average numbers of parasites per PV used in the figure were listed in a separate table. Data represent mean ± SD of n = 3 biological replicates. **b**, The Δ*alas*::*NLuc* mutant formed smaller plaques relative to WT::*NLuc* and Δ*alasALAS*::*NLuc* strains. The defect can be partially restored upon the addition of 300 μM ALA in the growth medium. Fifty plaques from 3 independent assays were measured using phase contrast light microscopy. Bar = 500 μm. Data represent mean ± SD. **c,** Concentration titration of ALA in restoring intracellular growth defects of the Δ*alas*::*NLuc* mutant. Parasite growth enhancement was observed when the medium was supplemented with 100 μM and 300 μM ALA and was restored to a greater extent with 300 μM ALA. Statistical significance was calculated by two-tailed unpaired Student’s *t*-test. *, *p*<0.05; **, *p*<0.01; ***, *p*<0.001; ****, *p*<0.0001; n.s., not significant.
(TIF)

S4 Fig *TgFECH* expression was regulated by a tetracycline-inducible TET-OFF system. **a,** Graphic description of gene epitope tagging and promoter swapping for the *TgFECH* gene. **b,** PCR verification of the integration of the 3xmyc tag and TET-OFF promoter into the *TgFECH* locus. Primers used in this study were indicated in the scheme.
(TIF)

S5 Fig *TgCPOX*- and *TgPPO*-deficient mutants exhibited defects in intracellular growth and plaque development. **a-b,** The heme-deficient parasites were grown in confluent HFFs and their luciferase activities were measured every 24 h for up to 96 h. The luciferase activities at 4 h post-infection were also determined for normalization. Error bars represent SEM. The assays were repeated in triplicate. **c-d,** The Δ*cpox* and Δ*ppo* mutants displayed smaller plaques than WT and the corresponding complementation strains. The plaques were allowed to develop in confluent HFFs for 7 days, without disturbance, before staining with crystal violet. Fifty plaques from 3 independent assays were measured using phase contrast light microscopy to compare their sizes. Bar = 500 μm. Data represent mean ± SD. Statistical significance was determined by two-tailed unpaired Student’s *t*-test. *, *p*<0.05; ****, *p*<0.0001; n.s., not significant.
(TIF)

S6 Fig Generation of heme-deficient yeast strains. **a,** Schematic illustration of the gene deletion strategy. **b,** PCR was used to verify the loss of the heme biosynthetic genes in yeast. Primers used in the study were labeled in the scheme. GOI, gene of interest; DRC, drug resistance cassette.
(TIF)

S7 Fig Determination of the IC_50_ values for succinylacetone (SA) in parasite growth. A luciferase-based assay was used for the determination. The IC_50_ values presented in the figure represent means ± SEM of n = 5 biological replicates.
(TIF)

S8 Fig Phylogenetic analysis of protoporphyrinogen oxidase (PPO). Neighbor-Joining consensus tree analysis of the relationships of 17 PPO family proteins derived from animals, plants, protozoa, fungi, and bacteria. Bootstrap values based on 10,000 replicates are shown. Accession numbers of protoporphyrinogen oxidase from individual species were listed in the parentheses. A human NADPH oxidase was also included as an outgroup for phylogeny construction. The closely related PPO orthologs were shaded in individual colors. Consensus bootstrap support (%) was labeled in the figure.
(TIF)

S9 Fig Screening of 11 commercially available PPO-targeting herbicides in the growth inhibition of *Toxoplasma* parasites. The five most potent inhibitors were identified with their IC_50_ values in the range of ~130–650 μM. Six inhibitors did not show significant inhibitions on parasite growth. The IC_50_ values for the top 5 known inhibitors were reported as means ± SEM of n = 3 biological replicates with 3 technical replicates each.
(TIF)

S10 Fig Quantitative PCR validation of *TgPPO* overexpression in the WT::*NLuc-pTub-TgPPO* strain. The qPCR assay was repeated in three biological replicates with three technical replicates each. Data shown in the figure were represented as mean ± SEM. *TgActin* was used as a normalization control. Statistical significance was calculated by two-tailed unpaired Student’s *t*-test. ***, *p*<0.001.
(TIF)

S11 Fig Evaluation of the toxicity of the synthesized oxadiazon derivatives in HFFs. An AlarmarBlue-based cell viability assay was used to evaluate the toxicity of oxadiazon and its derivatives. Triton X-100 was used as a positive control in the assay. Data represent means ± SEM of n = 3 biological replicates with 3 technical replicates each.
(TIF)

S12 Fig Extracellular heme did not rescue the intracellular growth of the Δ*cpox* parasites. A luciferase-based growth assay was used to measure the growth of the Δ*cpox* parasites in media containing or lacking 10 μM heme. The ALA-containing medium was used as a positive control. Data shown here represent means ± SEM of n = 3 biological replicates with 3 technical replicates each. Statistical significance was determined by two-tailed unpaired Student’s *t*-test. *, *p*<0.05; n.s., not significant.
(TIF)

S13 Fig Chemical structure validation of compound 1 by ^1^H and ^13^C NMR spectra. (TIF)

S14 Fig Chemical structure validation of compound 2 by ^1^H and ^13^C NMR spectra. (TIF)

S1 Text Description of the localization of TgUROD in the parasites, the generation of heme-deficient *Toxoplasma* strains, and plaque assay. (DOCX)

S2 Text Primers used in S2 Fig and S6 Fig. (DOCX)

S1 Table Ortholog search of heme biosynthetic genes in apicomplexan parasites using the Basic Local Alignment Search Tool (BLAST). Gene IDs listed in the table were identified by searching for orthologs of heme biosynthetic proteins in www.eupathdb.org.
(XLSX)

S2 Table *Toxoplasma* strains used in this study. (XLSX)

S3 Table Yeast strains used in this study. (XLSX)

S4 Table Primers used in this study. (XLSX)
