# Supplementary figures and images for: Toxoplasma gondii requires its plant-like heme biosynthesis pathway for infection
Source: PLoS Pathog. 2020 May 14;16(5):e1008499. doi: 10.1371/journal.ppat.1008499 (PMC7252677; doi:10.1371/journal.ppat.1008499)

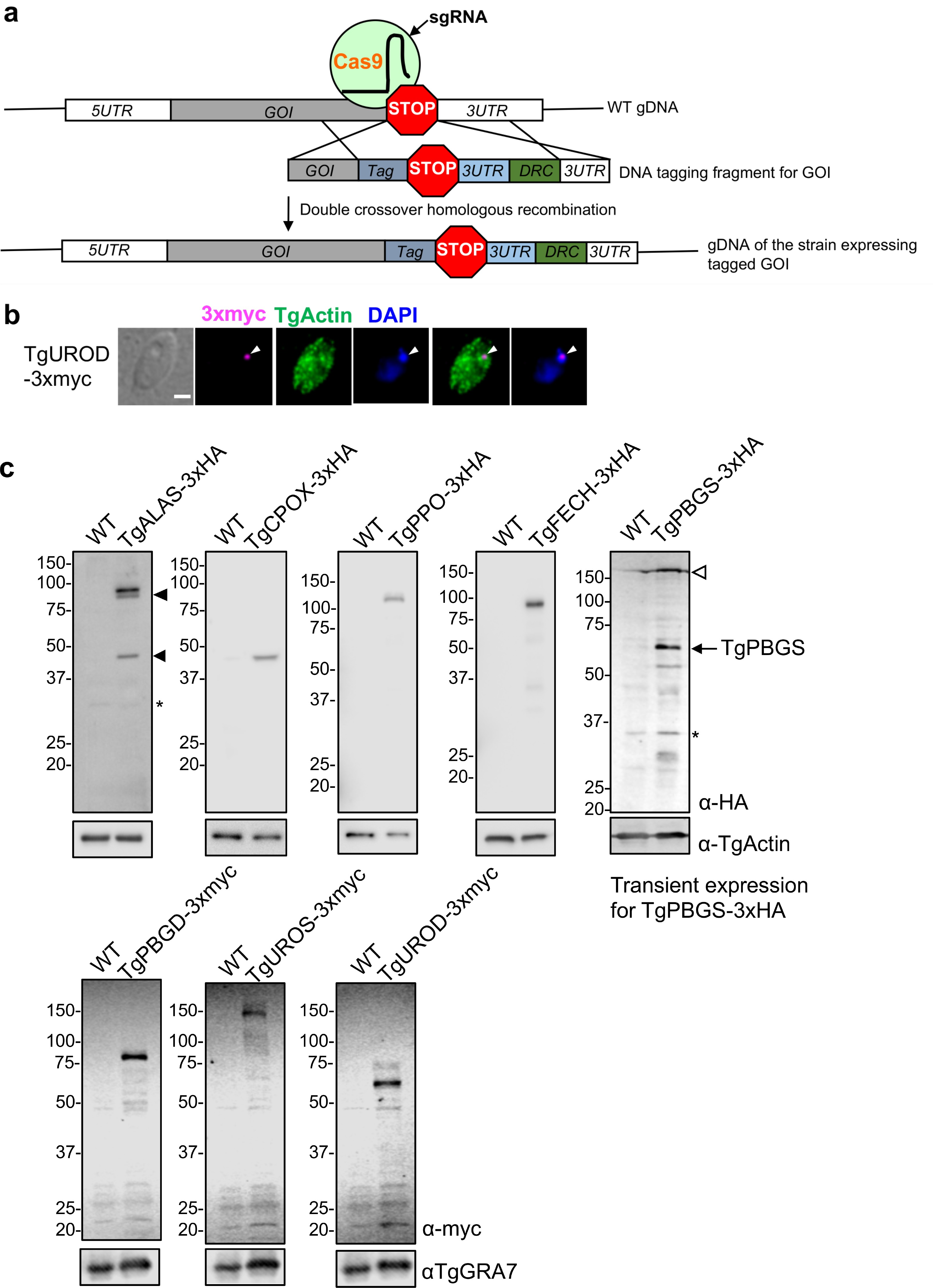

Supplement: S1 Fig — a, Schematic illustration of the endogenous gene epitope tagging in Toxoplasma. In brief, a 3xHA or 3xmyc tag was fused at the C-termini of the genes of interest using a CRISPR-Cas9-based cloning strategy. b, TgUROD was localized to the apicoplast, instead of the cytoplasm. TgActin was used as a cytoplasm marker. Bar = 2 μm. c, Immunoblotting analysis was used to confirm the expression of the epitope-tagged genes. The bands labeled with asterisks were derived from non-specific binding to antibodies. For TgALAS-3xHA, two protein fragments denoted by the filled arrowheads, migrating at ~80 kDa and ~40 kDa, represent the full-length and truncated TgALAS proteins, respectively. The TgPBGS gene only can be endogenously epitope-tagged in a transient manner. The protein lysate was purified from parasites that lysed immediately after transfection with the guide RNA expression construct and a TgPBGS-3xHA tagging DNA fragment. The guide RNA expression construct also expressed the 3xHA-tagged Cas9 proteins. Based on the predicted molecular weight, the band denoted by an unfilled arrowhead was derived from 3xHA-tagged Cas9. GOI, gene of interest; DRC, drug resistance cassette. (TIF) [file ppat.1008499.s001.tif]

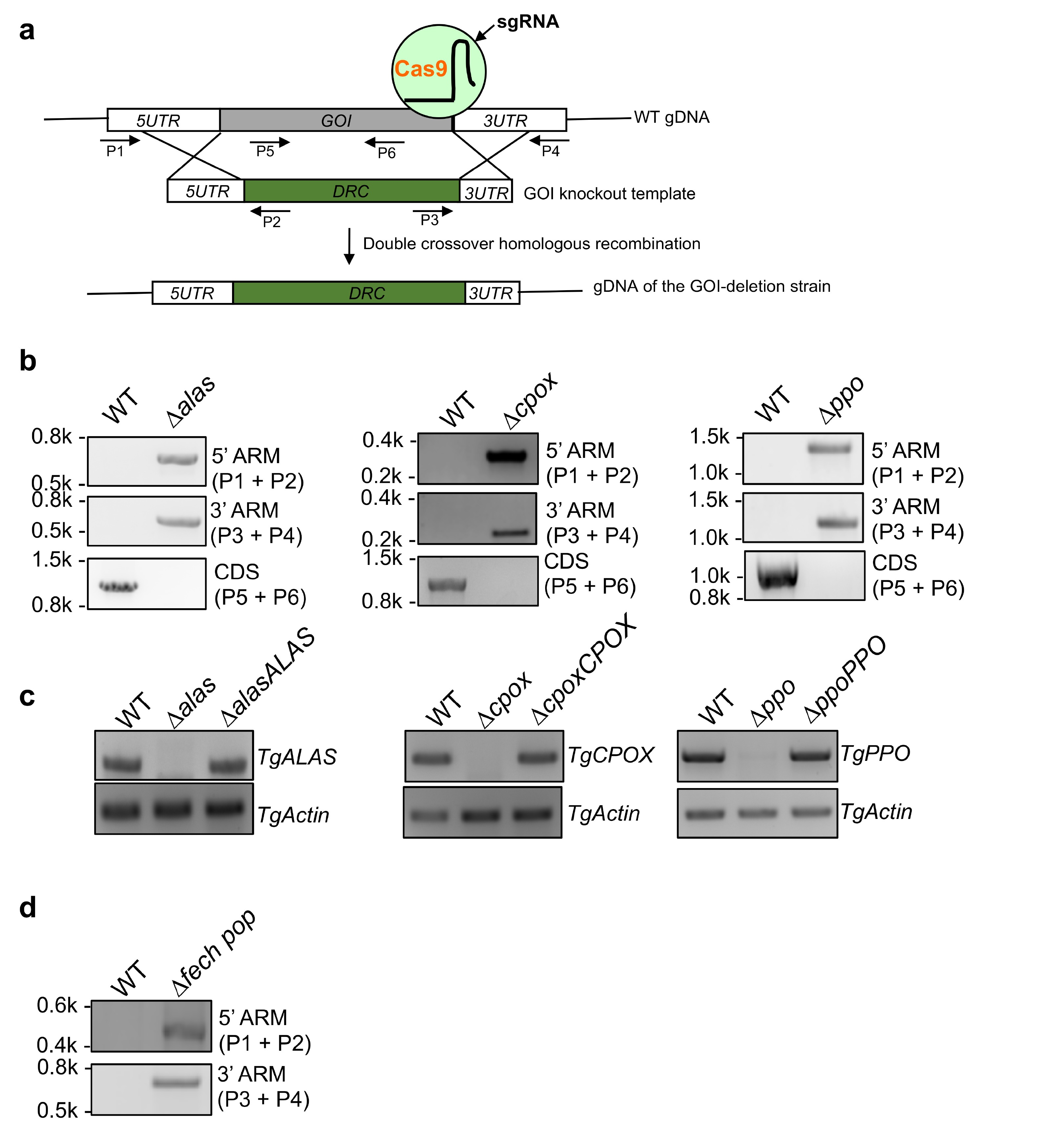

Supplement: S2 Fig — a, Schematic illustration of a general CRISPR-Cas9-based strategy for gene deletion in Toxoplasma. b, PCR confirmation of gene ablation. The genomic locations of the primers used in PCR amplification were indicated in the scheme. c, The loss of messenger RNA of the genes of interest was confirmed by reverse-transcription PCR (RT-PCR). d, The correct integration of the drug resistance cassette into the TgFECH locus during gene deletion was detected by PCR. However, the knockout parasites cannot be cloned probably due to its non-viability. GOI, gene of interest; DRC, drug resistance cassette. (TIF) [file ppat.1008499.s002.tif]

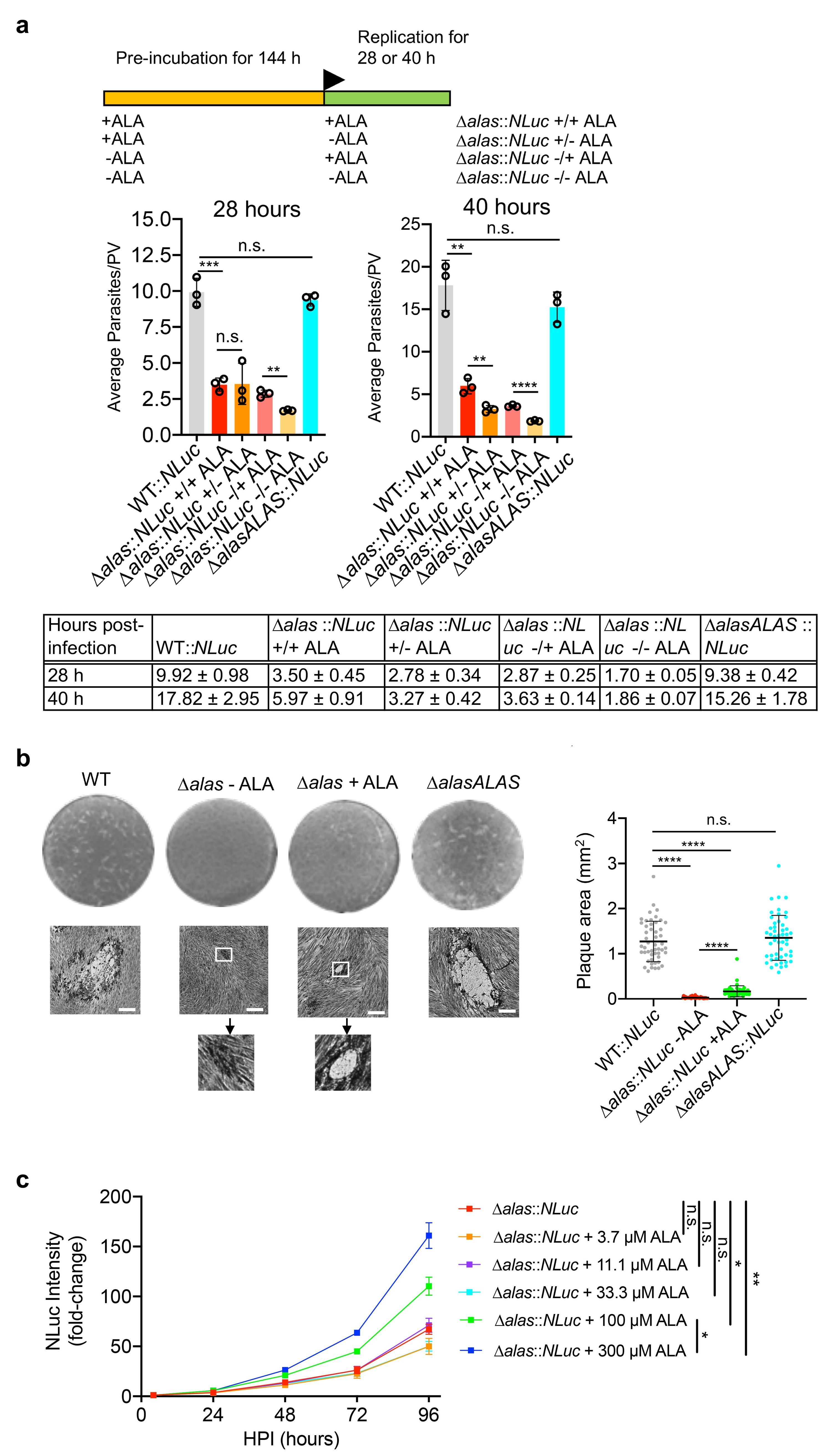

Supplement: S3 Fig — a, Replication comparison of the ALA-starved and non-starved Δalas::NLuc parasites in the media containing or lacking ALA. The average numbers of parasites per PV used in the figure were listed in a separate table. Data represent mean ± SD of n = 3 biological replicates. b, The Δalas::NLuc mutant formed smaller plaques relative to WT::NLuc and ΔalasALAS::NLuc strains. The defect can be partially restored upon the addition of 300 μM ALA in the growth medium. Fifty plaques from 3 independent assays were measured using phase contrast light microscopy. Bar = 500 μm. Data represent mean ± SD. c, Concentration titration of ALA in restoring intracellular growth defects of the Δalas::NLuc mutant. Parasite growth enhancement was observed when the medium was supplemented with 100 μM and 300 μM ALA and was restored to a greater extent with 300 μM ALA. Statistical significance was calculated by two-tailed unpaired Student’s t-test. *, p<0.05; **, p<0.01; ***, p<0.001; ****, p<0.0001; n.s., not significant. (TIF) [file ppat.1008499.s003.tif]

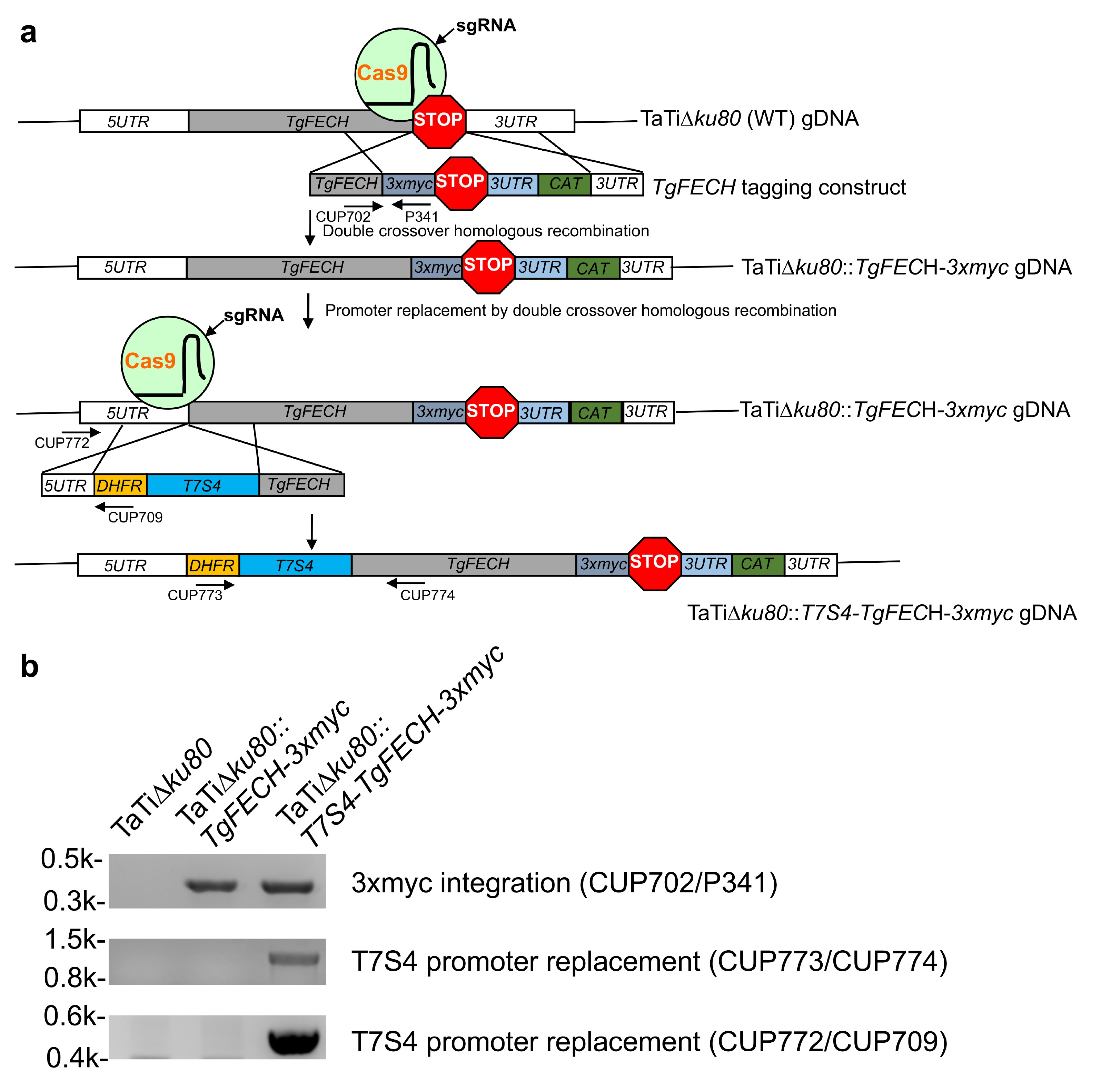

Supplement: S4 Fig — a, Graphic description of gene epitope tagging and promoter swapping for the TgFECH gene. b, PCR verification of the integration of the 3xmyc tag and TET-OFF promoter into the TgFECH locus. Primers used in this study were indicated in the scheme. (TIF) [file ppat.1008499.s004.tif]

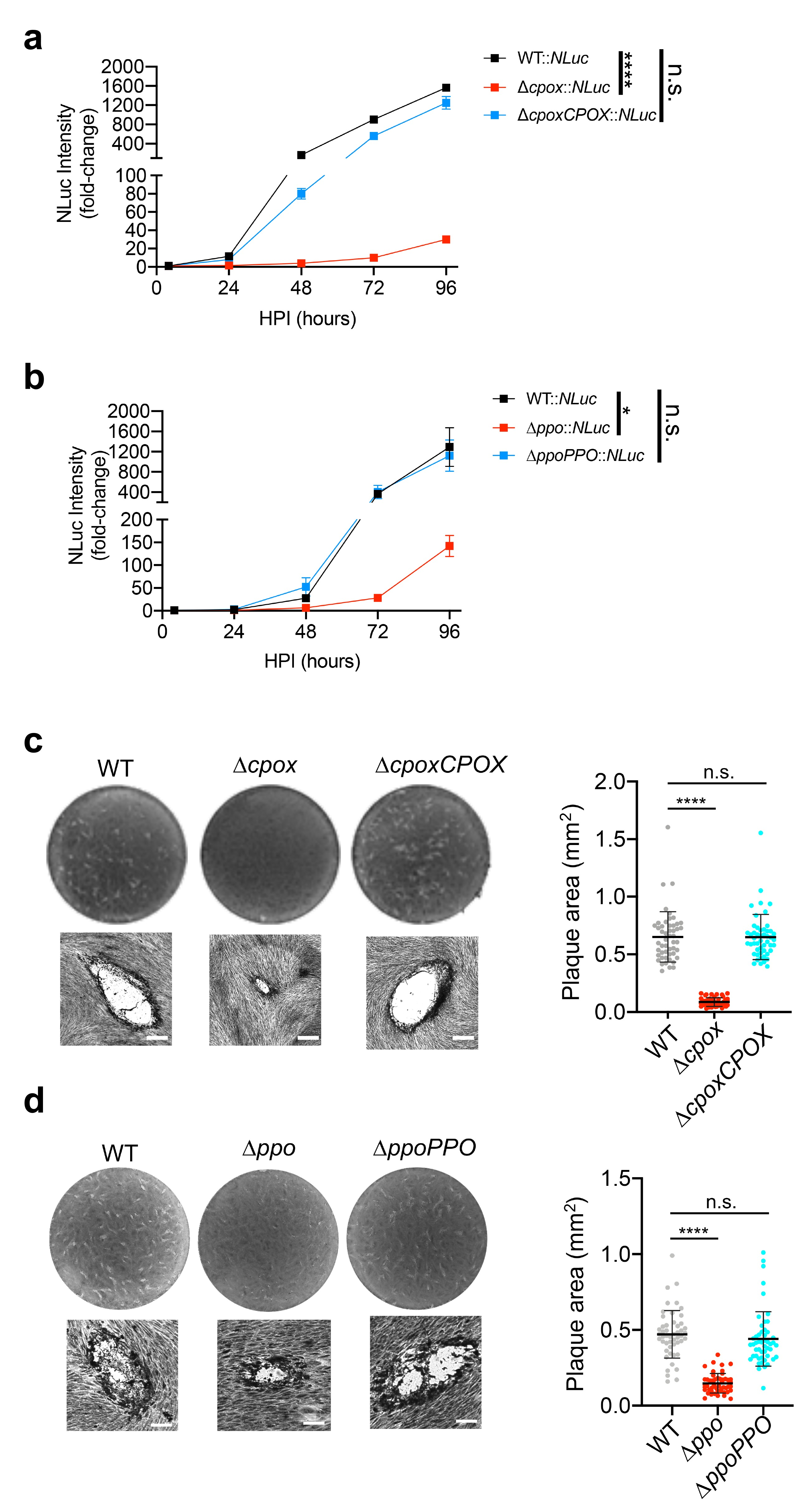

Supplement: S5 Fig — a-b, The heme-deficient parasites were grown in confluent HFFs and their luciferase activities were measured every 24 h for up to 96 h. The luciferase activities at 4 h post-infection were also determined for normalization. Error bars represent SEM. The assays were repeated in triplicate. c-d, The Δcpox and Δppo mutants displayed smaller plaques than WT and the corresponding complementation strains. The plaques were allowed to develop in confluent HFFs for 7 days, without disturbance, before staining with crystal violet. Fifty plaques from 3 independent assays were measured using phase contrast light microscopy to compare their sizes. Bar = 500 μm. Data represent mean ± SD. Statistical significance was determined by two-tailed unpaired Student’s t-test. *, p<0.05; ****, p<0.0001; n.s., not significant. (TIF) [file ppat.1008499.s005.tif]

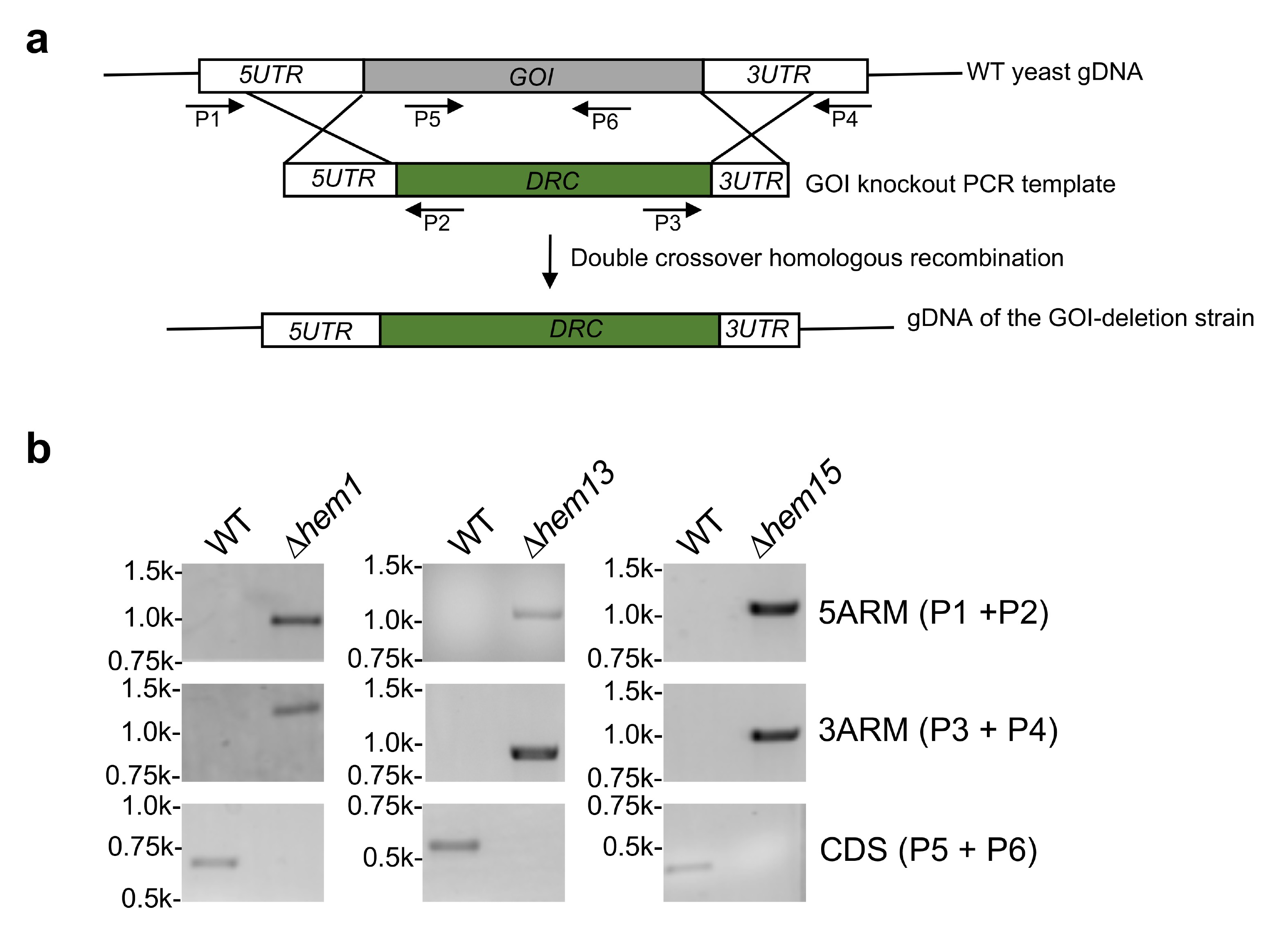

Supplement: S6 Fig — a, Schematic illustration of the gene deletion strategy. b, PCR was used to verify the loss of the heme biosynthetic genes in yeast. Primers used in the study were labeled in the scheme. GOI, gene of interest; DRC, drug resistance cassette. (TIF) [file ppat.1008499.s006.tif]

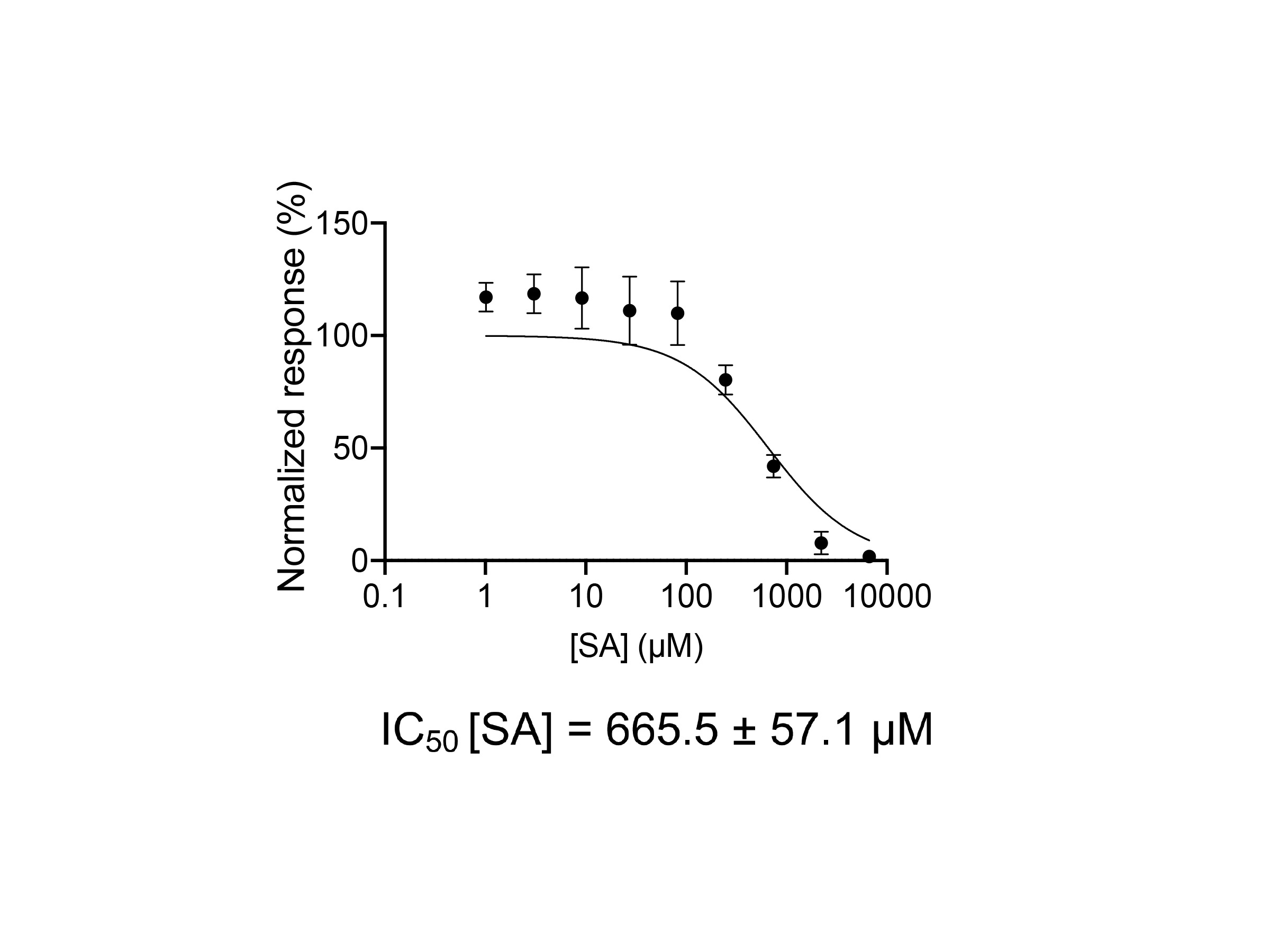

Supplement: S7 Fig — A luciferase-based assay was used for the determination. The IC50 values presented in the figure represent means ± SEM of n = 5 biological replicates. (TIF) [file ppat.1008499.s007.tif]

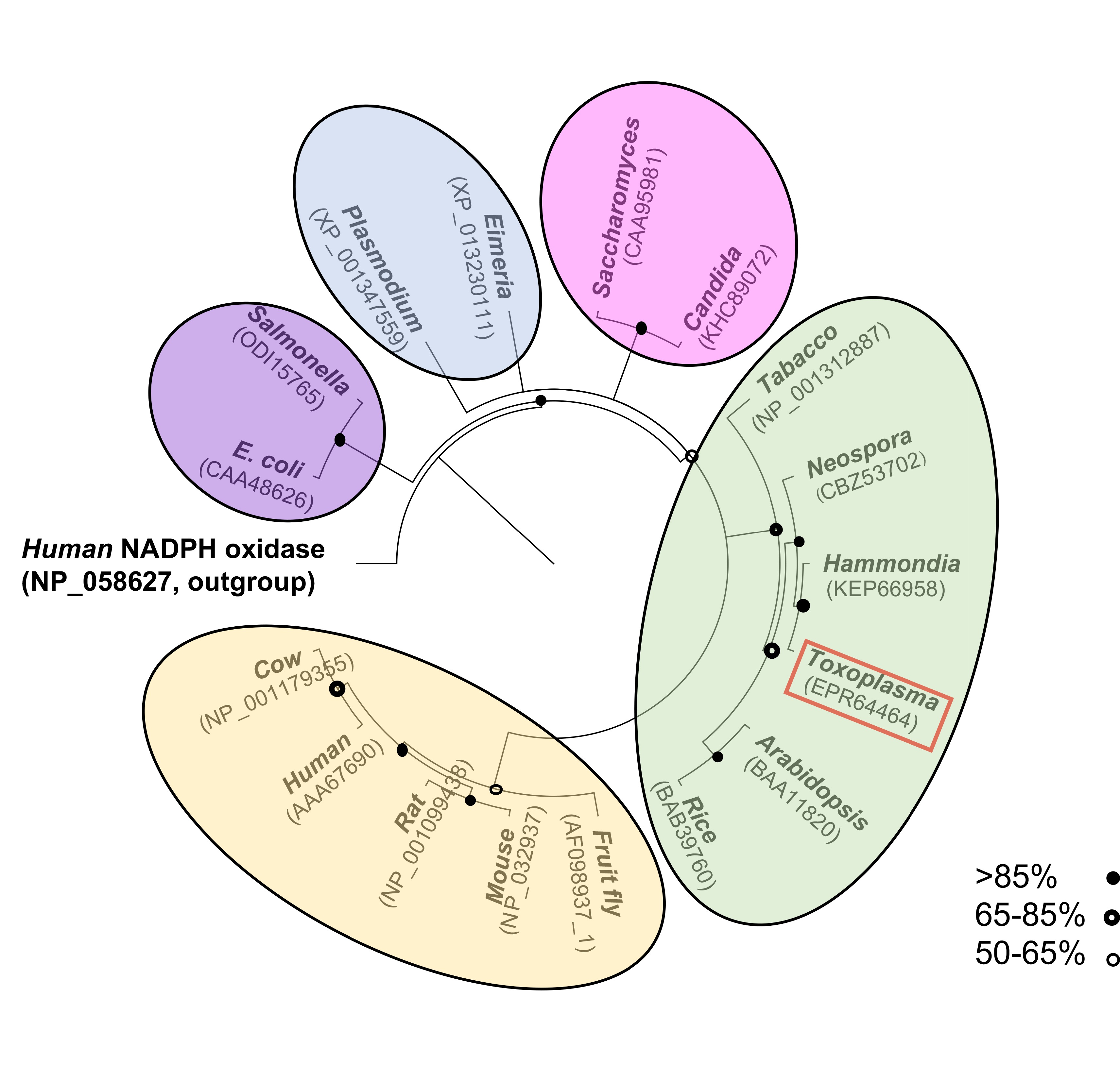

Supplement: S8 Fig — Neighbor-Joining consensus tree analysis of the relationships of 17 PPO family proteins derived from animals, plants, protozoa, fungi, and bacteria. Bootstrap values based on 10,000 replicates are shown. Accession numbers of protoporphyrinogen oxidase from individual species were listed in the parentheses. A human NADPH oxidase was also included as an outgroup for phylogeny construction. The closely related PPO orthologs were shaded in individual colors. Consensus bootstrap support (%) was labeled in the figure. (TIF) [file ppat.1008499.s008.tif]

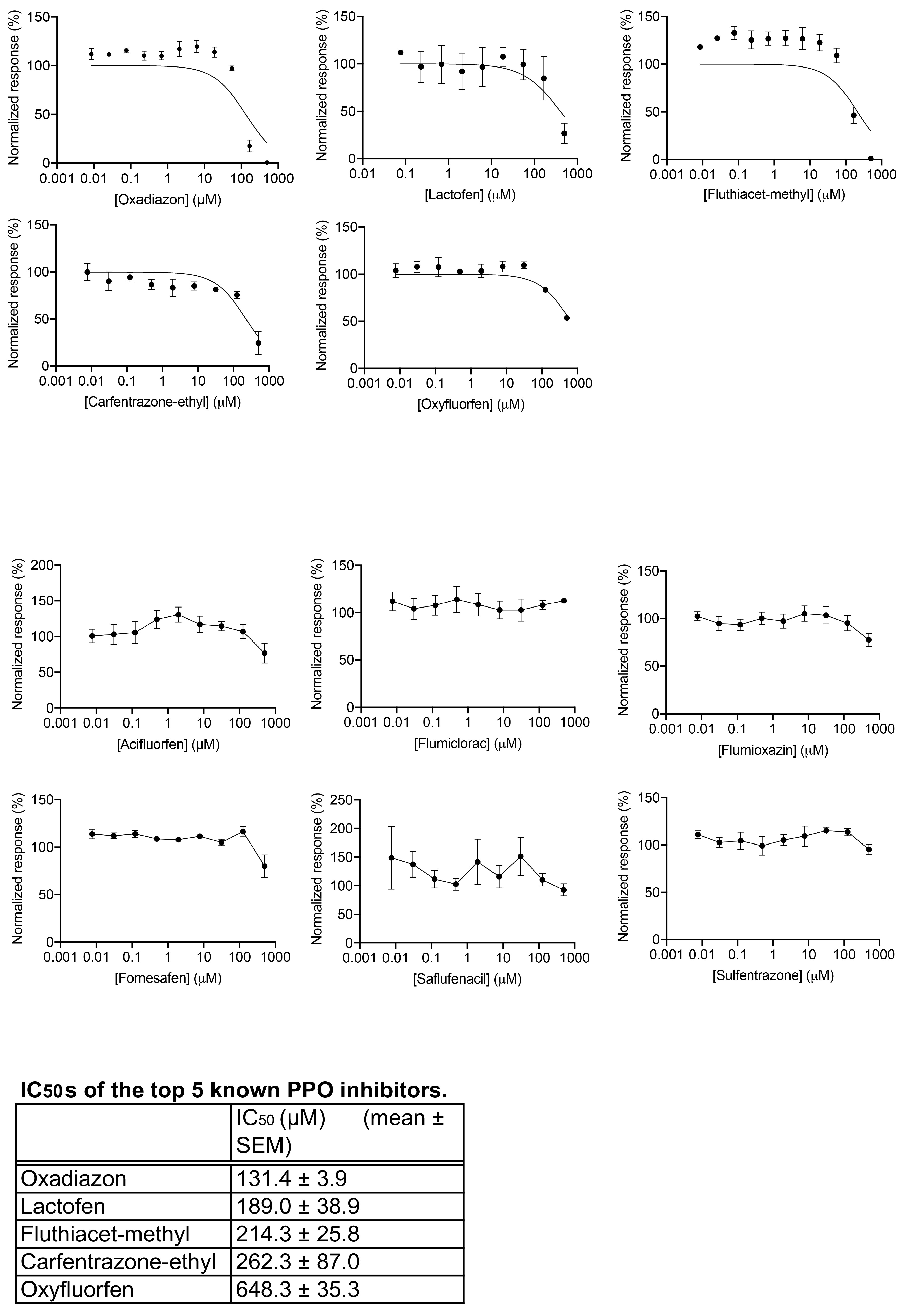

Supplement: S9 Fig — The five most potent inhibitors were identified with their IC50 values in the range of ~130–650 μM. Six inhibitors did not show significant inhibitions on parasite growth. The IC50 values for the top 5 known inhibitors were reported as means ± SEM of n = 3 biological replicates with 3 technical replicates each. (TIF) [file ppat.1008499.s009.tif]

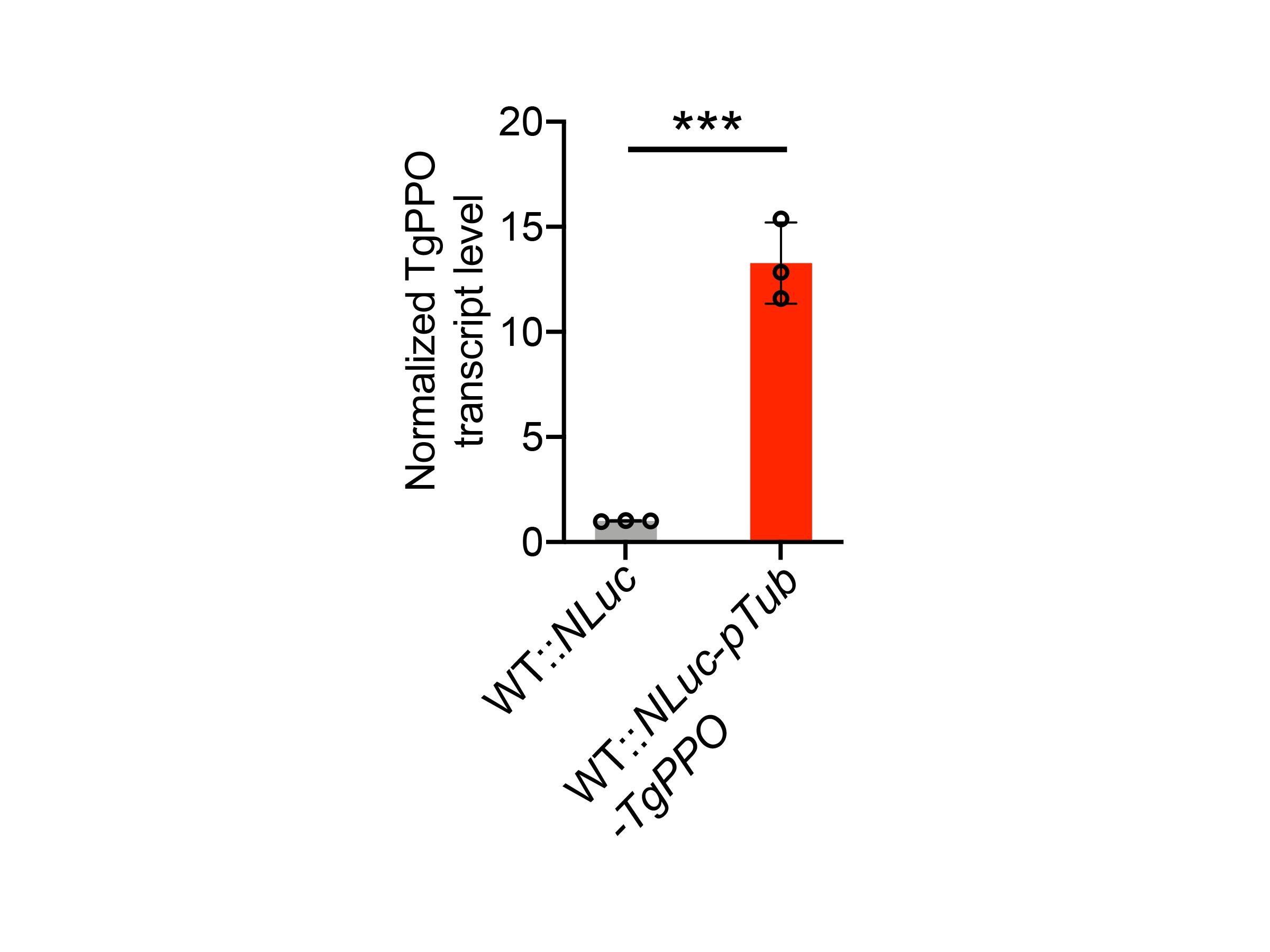

Supplement: S10 Fig — The qPCR assay was repeated in three biological replicates with three technical replicates each. Data shown in the figure were represented as mean ± SEM. TgActin was used as a normalization control. Statistical significance was calculated by two-tailed unpaired Student’s t-test. ***, p<0.001. (TIF) [file ppat.1008499.s010.tif]

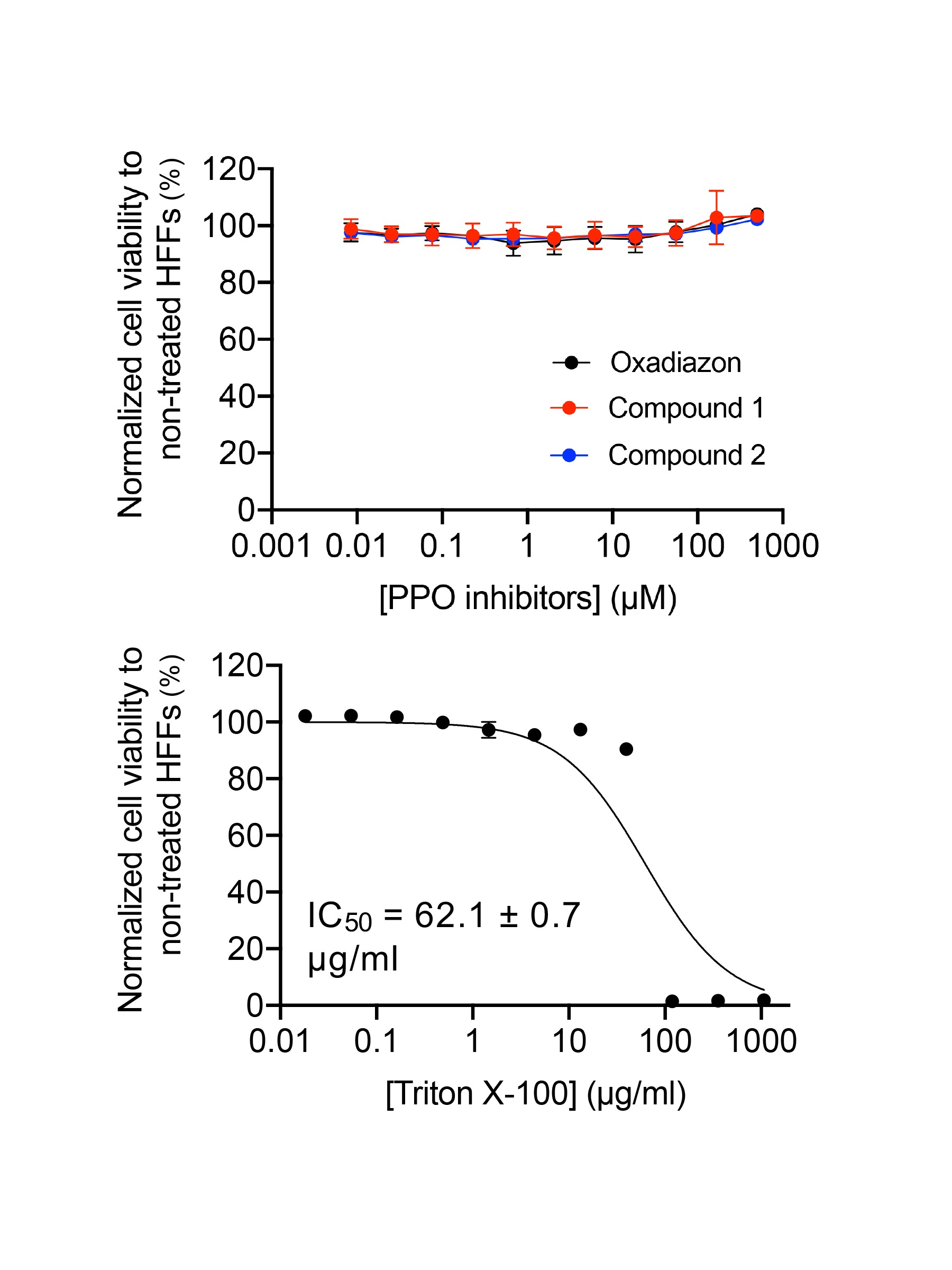

Supplement: S11 Fig — An AlarmarBlue-based cell viability assay was used to evaluate the toxicity of oxadiazon and its derivatives. Triton X-100 was used as a positive control in the assay. Data represent means ± SEM of n = 3 biological replicates with 3 technical replicates each. (TIF) [file ppat.1008499.s011.tif]

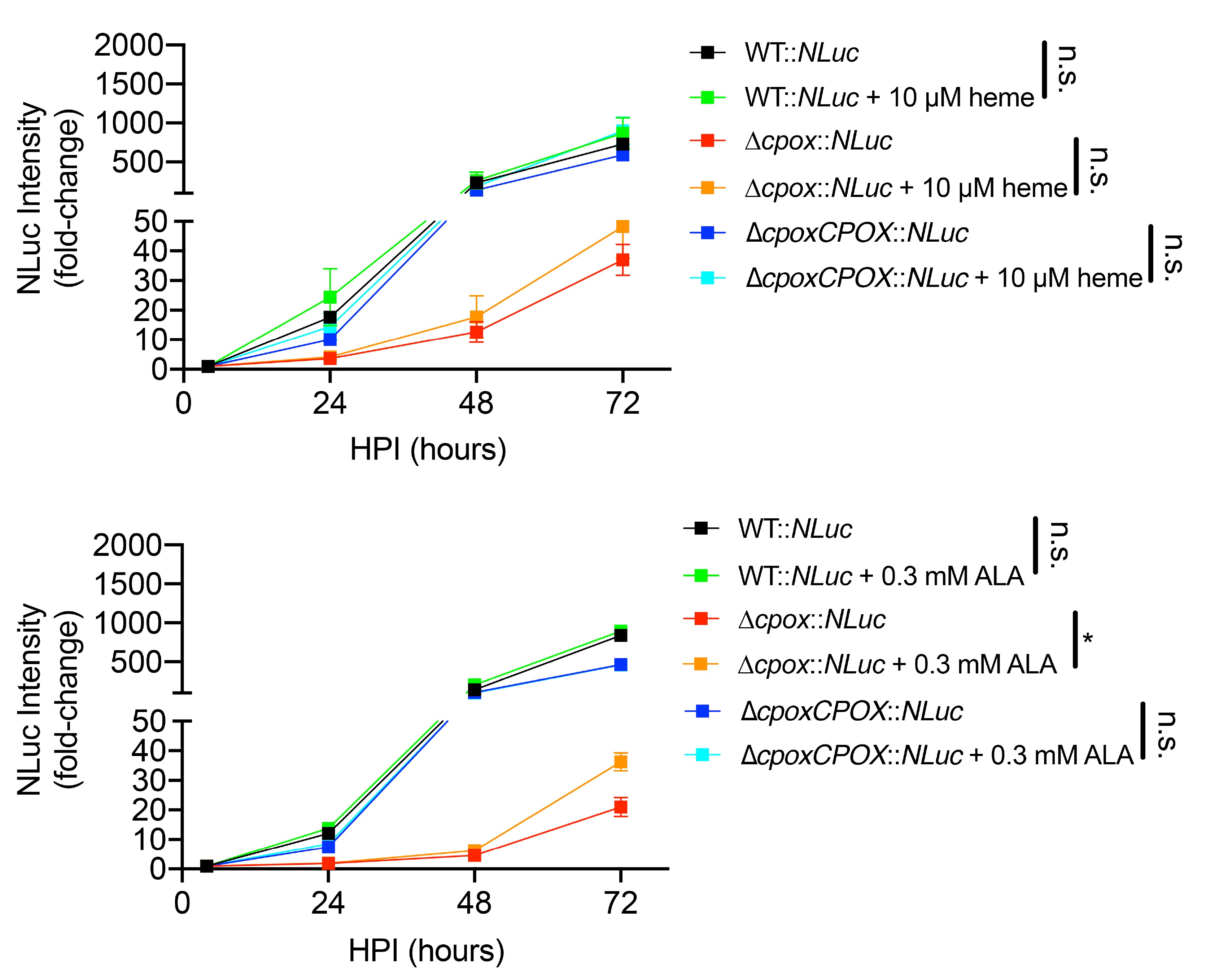

Supplement: S12 Fig — A luciferase-based growth assay was used to measure the growth of the Δcpox parasites in media containing or lacking 10 μM heme. The ALA-containing medium was used as a positive control. Data shown here represent means ± SEM of n = 3 biological replicates with 3 technical replicates each. Statistical significance was determined by two-tailed unpaired Student’s t-test. *, p<0.05; n.s., not significant. (TIF) [file ppat.1008499.s012.tif]

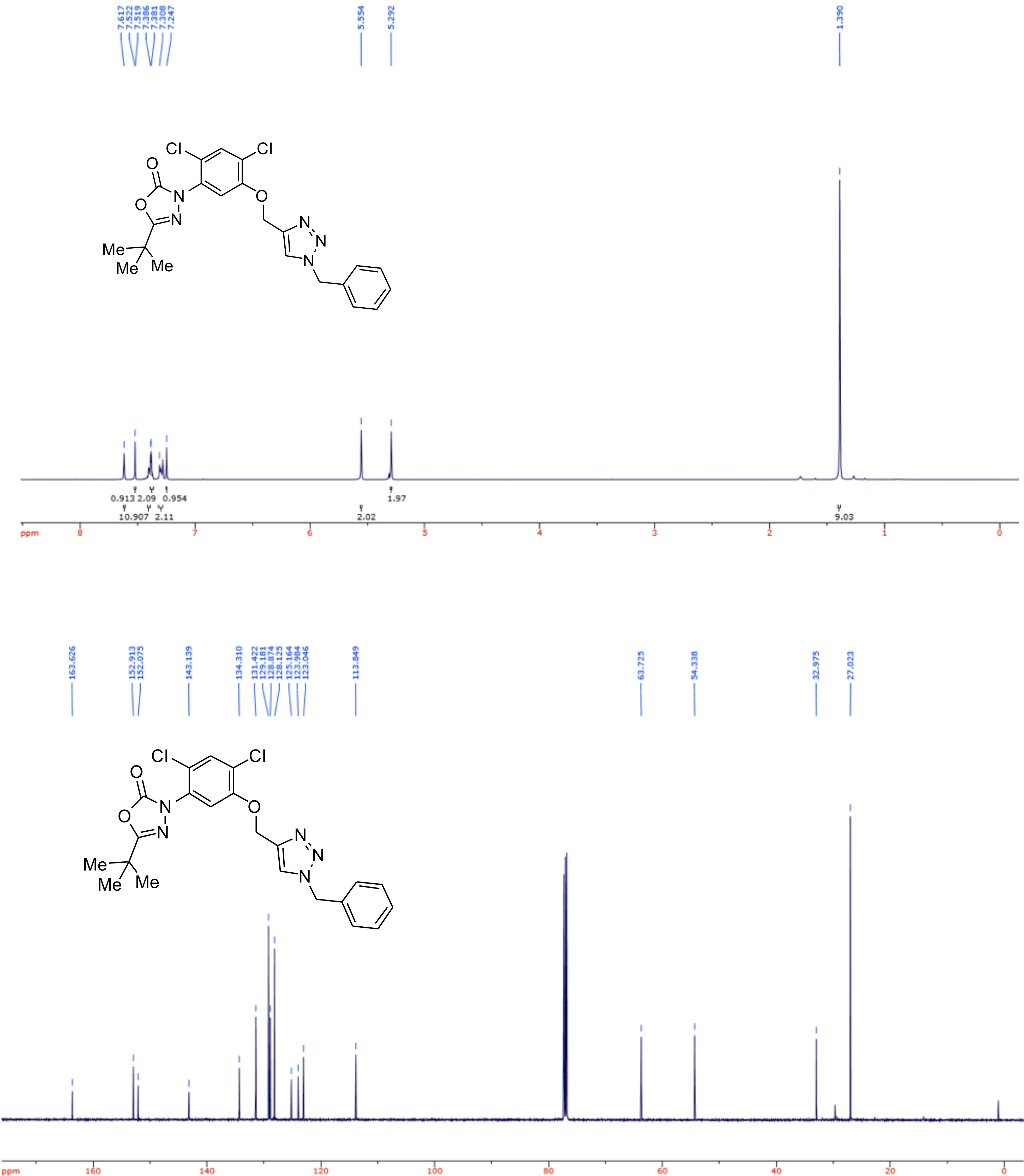

Supplement: S13 Fig — (TIF) [file ppat.1008499.s013.tif]

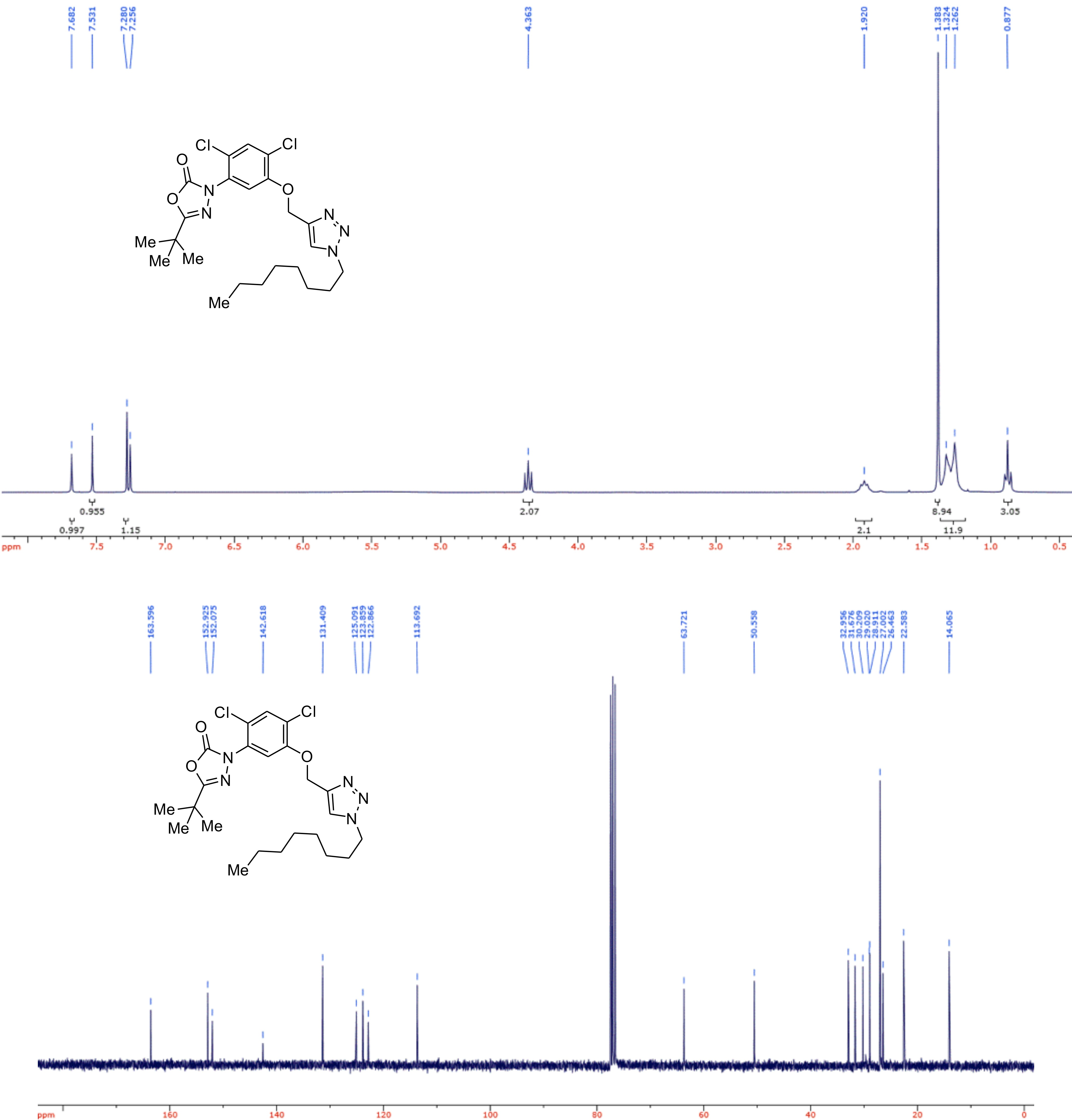

Supplement: S14 Fig — (TIF) [file ppat.1008499.s014.tif]
