# Supplementary material for: Toxoplasma gondii requires its plant-like heme biosynthesis pathway for infection
Source: PLoS Pathog. 2020 May 14;16(5):e1008499. doi: 10.1371/journal.ppat.1008499 (PMC7252677; doi:10.1371/journal.ppat.1008499)
Supplement: S1 Text — (DOCX) [file ppat.1008499.s015.docx]

**S1 Text. Description of the localization of TgUROD in the parasites, the generation of heme-deficient *Toxoplasma* strains, and plaque assay.**

**1. TgUROD is an apicoplast-localizing enzyme**

A previous study of overexpression of TgUROD revealed its subcellular location in the cytoplasm of the parasites [1]. By endogenous gene tagging, we determined its subcellular location in the apicoplast (Fig 1b and S1b Fig). Therefore, TgCPOX is the only cytosolic enzyme in the parasite’s heme biosynthetic pathway.

**2. Generation of the heme-deficient *Toxoplasma* strains**

We used CRISPR-Cas9-based genome editing tools to ablate the entire coding region, including introns and exons of 3 heme biosynthetic genes: *TgALAS*, *TgCPOX*, and *TgPPO*. Using PCR, we confirmed the removal of the endogenous genes and the integration of the drug resistance cassette (S2b Fig). Additionally, the total RNA from the knockouts, their parental strains, and the corresponding complementation strains were purified and tested by RT-PCR to confirm the loss of their messenger RNAs in the knockouts and the restored expression in the complementation strains (S2c Fig). We used a similar strategy to remove the *TgFECH* coding sequence in the parasites. After two rounds of drug selection, the integration of the bleomycin cassette into the *TgFECH* locus was detected by PCR (S2d Fig). However, we were unable to isolate the correct knockout clone from the population, suggesting that the *TgFECH* location in the genome is accessible for homologous recombination; however, *TgFECH* is required for parasite growth.

**3. The heme-deficient *Toxoplasma* parasites form small plaques.**

The heme-deficient parasites were used to infect confluent HFFs in 6-well plates. The plates were incubated at 37ºC with 5% CO_2_ for 7 days before plaque development was observed by crystal violet staining. The sizes of the plaques formed by ∆*cpox* and ∆*ppo* were reduced by ~90% and ~70%, respectively, compared to WT parasites (S5c and S5d Fig). For the *TgALAS*-deficient parasites, we grew the parasites in media containing or lacking ALA. The addition of extracellular ALA improved parasite growth. In the absence of ALA, the plaques formed by ∆*alas*::*NLuc* parasites showed small dark regions. In the absence and presence of ALA, the plaque areas of ∆*alas*::*NLuc* parasites were reduced by ~98% and 87%, respectively, relative to WT parasites (S3b Fig). Given that the replication rates of ∆*cpox* and ∆*ppo* were only reduced by ~75% and 50%, respectively, compared to WT parasites (Fig 2e and 2f), these findings suggest that the heme-deficient parasites may display defects in other steps of the parasite’s lytic cycle, such as invasion and egress.

**References**

1. Wu B. Heme biosynthetic pathway in apicomplexan parasites. PhD Dissertation, University of Pennsylvania. 2006.
