# Supplementary material for: Toxoplasma gondii requires its plant-like heme biosynthesis pathway for infection
Source: PLoS Pathog. 2020 May 14;16(5):e1008499. doi: 10.1371/journal.ppat.1008499 (PMC7252677; doi:10.1371/journal.ppat.1008499)
Supplement: S2 Text — (DOCX) [file ppat.1008499.s016.docx]

**S2 TEXT. Primers used in S2 Fig and S6 Fig.**

**1. Primers used in *Toxoplasma* knockout generation in S2 Fig.**

For ∆*alas*::*NLuc*, P1, CUP41; P2, P414; P3, P415; P4, CUP42; P5, CUP43; P6, CUP44.

For ∆*cpox*, P1, P467; P2, P414; P3, P415; P4, P468; P5, CUP223; P6, CUP224.

For ∆*ppo*, P1, CUP241; P2, P414; P3, P415; P4, CUP242; P5, CUP239; P6, CUP240.

For ∆*fech*, P1, CUP363; P2, P414; P3, P415; P4, CUP364.

**2. Primers used in yeast knockout generation in S6 Fig.**

For ∆*hem1*, P1, CUP554; P2, CUP302; P3, CUP303; P4, CUP555; P5, CUP561; P6, CUP560.

For ∆*hem13*, P1, CUP300; P2, CUP302; P3, CUP303; P4, CUP301; P5, CUP727; P6, CUP728.

For ∆*hem15*, P1, CUP705; P2, CUP302; P3, CUP303; P4, CUP706; P5, CUP703; P6, CUP704.

The CUP292 and CUP293 primers were used to test the introduction of the *Toxoplasma* orthologs in the trans-genera complementation strains.
